# *Plesiomonas shigelloides*, an Atypical Enterobacterales with a *Vibrio*-Related Secondary Chromosome

**DOI:** 10.1093/gbe/evac011

**Published:** 2022-01-25

**Authors:** Yazid Adam, Pierre Brezellec, Elena Espinosa, Amelie Besombes, Delphine Naquin, Evelyne Paly, Christophe Possoz, Erwin van Dijk, Francois-Xavier Barre, Jean-Luc Ferat

**Affiliations:** 1 Université Paris-Saclay, CEA, CNRS, Institute for Integrative Biology of the Cell (I2BC), Gif-sur-Yvette, France; 2 Université de Versailles Saint Quentin, Versailles, France; 3 Atelier de Bioinformatique, UMR 7205 ISYEB, CNRS-MNHN-UPMC-EPHE, Muséum d'Histoire Naturelle, Paris, France

**Keywords:** *Plesiomonas shigelloides*, Vibrionales, Enterobacterales, RctB, chromid, SeqA, MatP

## Abstract

About 10% of bacteria have a multichromosome genome with a primary replicon of bacterial origin, called the chromosome, and other replicons of plasmid origin, the chromids. Studies on multichromosome bacteria revealed potential points of coordination between the replication/segregation of chromids and the progression of the cell cycle. For example, replication of the chromid of Vibrionales (called Chr2) is initiated upon duplication of a sequence carried by the primary chromosome (called Chr1), in such a way that replication of both replicons is completed synchronously. Also, Chr2 uses the Chr1 as a scaffold for its partition in the daughter cells. How many of the features detected so far are required for the proper integration of a secondary chromosome in the cell cycle? How many more features remain to be discovered? We hypothesized that critical features for the integration of the replication/segregation of a given chromid within the cell cycle program would be conserved independently of the species in which the chromid has settled. Hence, we searched for a chromid related to that found in Vibrionales outside of this order. We identified one in *Plesiomonas shigelloides*, an aquatic and pathogenic enterobacterium that diverged early within the clade of Enterobacterales. Our results suggest that the chromids present in *P. shigelloides* and Vibrionales derive from a common ancestor. We initiated in silico genomic and proteomic comparative analyses of *P. shigelloides*, Vibrionales, and Enterobacterales that enabled us to establish a list of features likely involved in the maintenance of the chromid within the host cell cycle.

Significance

Vibrionales and Enterobacterales are two closely related orders that derive from a common ancestor. Although Vibrionales are multichromosome species, Enterobacterales are known to be monochromosome bacteria. What are the features and factors needed to ensure the sustainability of multiple chromosomes in a cell? We addressed this question by searching and identifying an Enterobacterium with multiple chromosomes, *Plesiomonas shigelloides*, and by carrying out a comparative analysis of its genome and proteome with those of the monochromosome Enterobacterales and the multichromosome Vibrionales.

## Introduction

Most bacteria are monochromosome bacteria, that is, they carry their core-genome on a unique bacterial chromosome. On the other hand, a fraction of bacteria (called multichromosome bacteria) have a composite genome in which the core-genome is split in two or more replicons. The larger replicon possesses a replication initiation setting (replication origin and initiator protein) homologous to that found in monochromosome bacteria, whereas the other (usually smaller) replicons are of plasmid origin. They are referred to as chromids. A major criterium distinguishing chromids from plasmids is that the latter are erratically distributed among bacteria whereas chromids are systematically present in all the strains of a given clade, which can be a species, a genus, or an order, like the Vibrionales. Other criteria for qualifying a replicon as a chromid were also proposed such as the transfer of core-genome genes on the chromid, some of which might even be essential (diCenzo and Finan 2017).

Vibrionales is an order of gammaproteobacteria that regroups aquatic bacteria, among which the model organism *Vibrio cholerae*, the causative agent of cholera. The genome of Vibrionales is split into a primary circular chromosome (Chr1) and a smaller circular chromid, referred to as the secondary chromosome (Chr2) (Trucksis et al. 1998; Yamaichi et al. 1999; Kirkup et al. 2010). *Vibrionales* are closely related to Enterobacterales and both orders emerged from a last common ancestor (LCA)*.* In contrast, Enterobacterales regroups monochromosome bacteria, among which the model organism *Escherichia coli*, suggesting that the Chr2 of Vibrionales evolved from a plasmid that was either acquired early in the history of the Vibrionales or earlier even, in the LCA and lost secondarily in the Enterobacterales.

Based on studies of *V. cholerae*, the persistence of Chr2 in Vibrionales (and prospectively of chromids in general) was associated with the selection of several genetic features ensuring a tight control of its initiation of replication and a finely tuned coordination of its replication/segregation within the host cell cycle (Ramachandran et al. 2016; Espinosa et al. 2017).

In Vibrionales, replication of Chr2 is initiated once and only once per cell cycle. Replication is triggered at *ori2* (an iteron-based origin of replication), whose organization is similar to that found in F and P1 plasmids, and directed by RctB—a homolog of plasmid replication initiator proteins like RepA (Orlova et al. 2017). Replication of Chr2 occurs during that of Chr1 and such that the termination of the replication of Chr1 and Chr2 are synchronized (Rasmussen et al. 2007). Chr2 copy number is controlled through plasmid-like molecular mechanisms such as the negative control of *rctB* transcription and the handcuffing of newly synthesized *ori2* sisters by RctB dimers (Ramachandran et al. 2016). Chr2 replication initiation is also controlled by bacterial chromosome-like mechanisms. Some are specified by proteins of the Dam cohort (Brézellec et al. 2006). The affinity of RctB for the iterons is modulated by the Dam-dependent methylation of *ori2*, which might be sequestrated by SeqA after replication initiation, like the origin of replication of the bacterial chromosome (Lu et al. 1994). Finally, the perfect timing of Chr2 replication initiation is dictated by a short sequence located on the main chromosome (*crtS*), whose duplication triggers replication initiation of Chr2 (Baek and Chattoraj 2014; Val et al. 2016; Ramachandran et al. 2018). The same coupling seems to operate in all the *Vibrionales* (Kemter et al. 2018).

Chr2 segregation is driven by an essential partition machinery, *parAB2*, which uses Chr1 DNA as a scaffold to distribute newly replicated copies along the cell axis (Yamaichi et al. 2007). Tracking of several loci along Chr1 and Chr2 revealed that the terminus region of the two replicons, *ter1* and *ter2*, are synchronously recruited at midcell before duplication, whereas the rest of the genome is already segregated (David et al. 2014). Sister copies of *ter1* and *ter2* are maintained at midcell before cell division by MatP (Demarre et al. 2014), which is part of the *dam* cohort (Brézellec et al. 2006). In *E. coli*, MatP was shown to exert its function by interacting with specific sequence motifs, the *matS* sites, which are only present in the terminus region of the chromosome (Mercier et al. 2008). The positioning of sister copies of *ter1* and *ter2* at midcell is important for the resolution of RecA-induced chromosome dimers after replication. A site-specific recombination machinery (Xer) acts at specific chromosomal sites, *dif1* and *dif2* located in *ter1* and *ter2*, respectively, through a direct contact with the cell division protein and DNA translocase, FtsK (Val et al. 2008). FtsK is directed toward *dif1* and *dif2* by KOPS sequence motifs located on both arms of the two replicons (Val et al. 2008). The positioning of sister copies of *ter2* at midcell is important for the proper licensing of cell division at the end of the replication/segregation cycle because binding sites for an inhibitor of cell division, SlmA, are found on Chr2 except for its terminus region (Bernhardt and de Boer 2005; Galli et al. 2016). Thus, a key point of the cell cycle coordination appears to be the synchronized recruitment of the termini of both Chr1 and Chr2 at midcell.

Which of the genetic features identified as critical for the maintenance of Chr2 in *V. cholerae* are common adaptation traits of chromids? We addressed this question by searching for species outside of *Vibrionales* and carrying a chromid that evolved from the same ancestor plasmid that gave rise to Chr2 of *Vibrionales*. We assumed that chromids with common ancestry and orthologous initiator proteins (i.e., initiator proteins with common and identifiable protein domains) would have been integrated within the cell cycle program of the host through the acquisition of similar adaptive modifications that are as many critical features of the replication/segregation of chromids. Hence, we searched RctB-like proteins outside Vibrionales. We identified a RctB-chromid in *Plesiomonas shigelloides*. We established that *P. shigelloides* is an orphan species of Enterobacterales in which the genome is composed of a main chromosome with a bacterial origin of replication (*ori*^Chm^) and a chromid whose replication origin (*ori*^Chd^) is structured like those found on Chr2 of Vibrionales. The origin of replication of the chromid of *P. shigelloides* is rich in GATC sequences like the origin of the Chr2 or Vibrionales and its replication terminus region (*ter*^Chd^) contains numerous MatP-binding sites like the terminus of the Chr2 or Vibrionales. A *dif* site was identified in *ter*^Chd^, and both replichores are enriched in polar KOPS pointing toward this site. Thus, several adaptative traits proposed to be important for the maintenance of chromids were detected in the chromid of *P. shigelloides*. Interestingly, we detected motifs typical of Vibrionales in the SeqA (PsSeqA) and MatP (PsMAtP) proteins of *P. shigelloides*, suggesting the acquisition of chromid-related adaptive features. Finally, we established a list of Vibrionales- and Enterobacterales-restricted protein domains, which are both represented in the domainome of *P. shigelloides*. We show that some of these domains were likely acquired or maintained due to the ecological niche of *P. shigelloides*.

### *Plesiomonas shigelloides* Contains a RctB-Chromid

The structure of the two central domains (2–3) of RctB is similar with, and common among, plasmid replication initiators (Orlova et al. 2017). Yet, these domains bear a Hidden Markov Model (HMM) signature distinctive enough in RctB to be diagnostic of the Vibrionales initiator protein (PF11826 or DUF3346) (supplementary fig. 1, Supplementary Material online). We searched and returned from the UniProtKB library other proteins carrying this domain. DUF3346 is present in the genome of several plasmids that proliferate in Vibrionales and interestingly in non-Vibrionales species. We returned two positive hits, one in *P.shigelloides* and the other in *Ferrimonas marina*. Yet, DUF3346 was detected in only one of the six strains of *Ferrimonas* available in UniProtKB. As the DUF3346-containing protein was not identified in all the strains of the clade, we excluded it from our analysis. In contrast, DUF3346 was identified in each of the four genomes of *Plesiomona*s deposited at the time of our analysis in the UniProtKB library. We confirmed the apparent ubiquitous presence of *rctB* in *P. shigelloides* by PCR amplification in nine strains of the species picked randomly in the *P.shigelloides* collection of Sylvain Brisse at the Pasteur Institute (Salerno et al. 2007). We further assessed that it corresponded to the presence of a *rctB*-replicon in each of these isolates by genome sequencing (Materials and Methods; supplementary fig. 5, Supplementary Material online).

We assembled the genome of strain 7A and used it as a matrix for the assembly of the *ori* region of all the other strains sequenced (Materials and Methods). The presumptive origin of replication of the RctB replicon corresponded to a large orf-free DNA sequence (∼1.6 kb) flanked on one side by *rctB* and on the other side by the *parAB* operon (supplementary fig. 2, Supplementary Material online). Careful scrutiny revealed a complex organization of the origin of replication, similar to that found in the Chr2 of Vibrionales. We identified two types of conserved iteron-like sequences distributed on each side of a DnaA box sequence (supplementary fig. 2, Supplementary Material online). Six 10 mer sequences [5′ CGGA(T/C)GGATC 3′] are located upstream of the *parAB* operon, whereas six 8 mer sequences [5′-TGGATCGT-3′] lay upstream of the *rctB*-like gene. Strikingly, the 8 mers are regularly scattered, like the 12 mers in the origin of replication of the Chr2 of the Vibrionales.

**Fig. 1:**
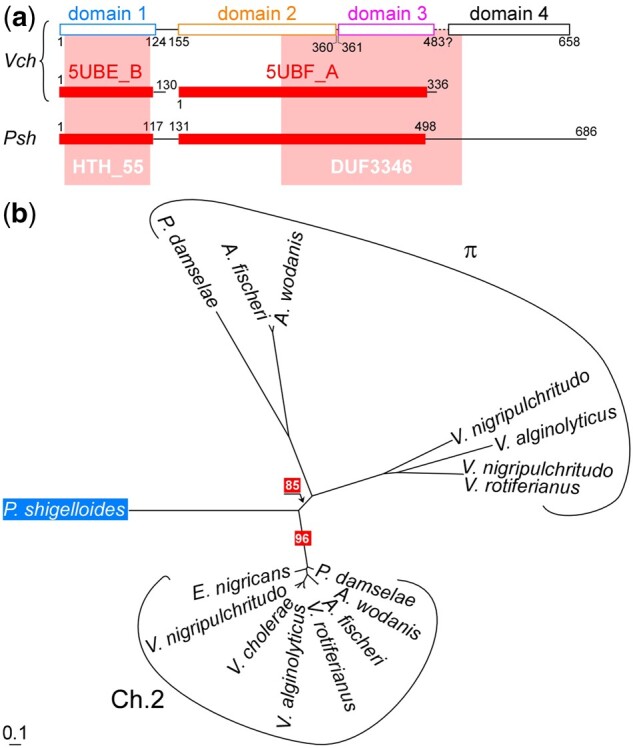
*Plesiomonas shigelloides* contains a RctB-replicon. (*a*) Architecture of RctB in four structural domains (d1–d4). The positions of the HMM profiles detected in RctB: HTH_55 (d1) and DUF3346 (d2–d3) are shaded in light red. The polypeptide fragments of the RctB proteins of *V. cholerae* that were crystallized are indicated (5UBE_B and 5UBF_A). The DUF3346-carrying sequences identified in *P. shigelloides* (*Psh*) is shown. Red rectangles delineate HMM homology with *V. cholerae* RctB. (*b*) RctB-based phylogenetic tree. The branch of *P. shigelloides* is distinct from those of the chromosome 2 (Chr2) or the plasmids (π) of Vibrionales. Relevant bootstrap scores and a scale indicative of the substitution frequency are provided. *Enterovibrio nigricans* DSM 22720, *Aliivibrio fischeri* MJ11, *Vibrio nigripulchritudo* FTn2, *Vibrio alginolyticus* HKB31, *Vibrio rotiferianus* AM7, *Photobacterium damselae* BST98, *Aliivibrio wodanis* AW0309160, *Vibrio cholerae* serotype O1 El Tor Inaba N16961, *Plesiomonas shigelloides* 7A.

A protein analysis was then carried out on *P. shigelloides* RctB. HHpred analysis revealed the presence of two Pfam domains: HTH_55 and DUF3346, which are diagnostic of structural domains 1 and 2–3 of RctB of Vibrionales, respectively (supplementary fig. 1, Supplementary Material online). The analysis predicted also a folding similar to that established for domains 1 and 2–3 of the RctB of *V. cholerae*, respectively (fig. 1*a*). Finally, we collected the RctB protein sequences of *P. shigelloides*, of plasmids and Chr2 of Vibrionales and compared them through a phylogenetic analysis (fig. 1*b*). The RctB protein identified in *Plesiomonas* forms an evolutive branch distinct of that of the Vibrionales Chr2 and plasmids. Altogether, these results indicates that *P. shigelloides* possesses a chromid, whose replication initiation machinery is similar to, but distinct from, the chromosome 2 of Vibrionales.

### *Plesiomonas shigelloides* Is an Enterobacterales

Over the last decades, *P.shigelloides* was alternately ranged in the Aeromonadales, the Vibrionales and finally in the Enterobacterales (Janda et al. 2016). The facts that *P. shigelloides* is found as a free-living organism in aquatic environments and as an opportunistic pathogen are likely explaining why its classification was challenging.

**Fig. 2:**
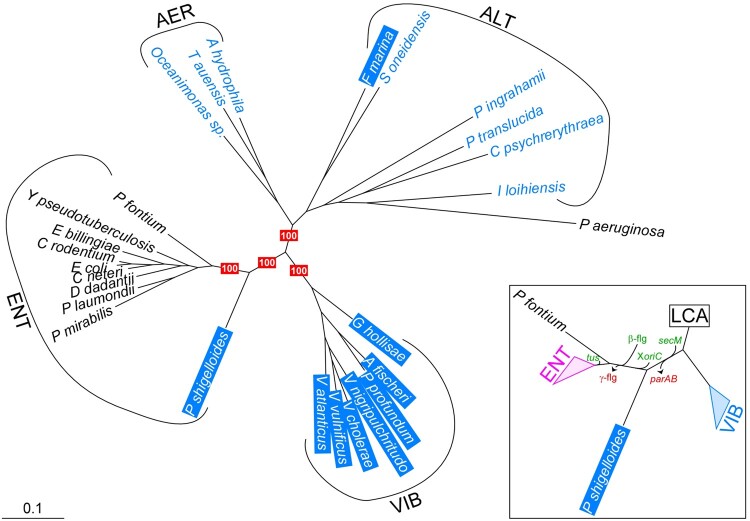
*Plesiomonas* is an atypical Enterobacterales. Phylogenetic tree based on a concatenation of DnaA, DnaB, DnaE, and DnaX protein sequences. Monophyletic clades corresponding to known orders of gammaproteobacteria are indicated: Enterobacterales (ENT), Vibrionales (VIB), Aeromonadales (AER), and Alteromonadales (ALT). *Pseudomonas aeruginosa* PAO1 was used as an outgroup. Species whose biotope is aquatic are in blue and those in which a RctB-replicon was detected are highlighted in blue. Relevant bootstrap scores and a scale indicative of the substitution frequency are provided. Some genomic modifications that occurred through evolution are reported in the inset. The acquisitions and losses of genes are in green and red, respectively. X*oriC* correspond to the recombination that occurred at *oriC* (supplementary fig. 2, Supplementary Material online). β-flg and γ-flg refer to the peritrichous and polar flagellar system, respectively (Ferreira et al. 2021). *Yersinia pestis*, *Proteus mirabilis* HI4320, *Pragia fontium*, *Plesiomonas shigelloides* 7A, *Photorhabdus laumondii* subsp. laumondii TT01, *Escherichia coli* K12, *Erwinia billingiae* Eb661, *Dickeya dadantii* 3937, *Citrobacter rodentium* ICC168, *Cedecea neteri*, *Vibrio vulnificus* YJ016, *Vibrio tasmaniensis* LGP32, *Vibrio nigripulchritudo*, *Vibrio mangrovi*, *Vibrio cholerae* serotype O1 El Tor Inaba N16961, *Photobacterium profundum* SS9, *Grimontia hollisae* CIP 101886, *Aliivibrio fischeri* ES114, *Shewanella oneidensis* MR-1, *Psychromonas ingrahamii* 37, *Pseudoalteromonas haloplanktis* TAC 125, *Idiomarina loihiensis* L2-TR, *Ferrimonas marina*, *Colwellia psychrerythraea* 34H, *Agarivorans albus* MKT 106, *Tolumonas auensis* TA4, *Oceanimonas* sp. GK1, *Aeromonas hydrophila* subsp. hydrophila NCIMB 9240, *Pseudomonas aeruginosa* PAO1.

The presence of a *rctB*-replicon, otherwise associated with Vibrionales, led us to reassess phylogenetically the rooting of *P. shigelloides* within the gammaproteobacteria. To this end, we concatenated protein sequences encoded by six house-keeping genes involved in replication (*dnaA*, *dnaE*), transcription (*mfd*, *rpoB*), metabolism (*purL*), and translation (*valS*). The phylogenic tree obtained shows that *P. shigelloides* is not included in the main clades of Enterobacterales and Vibrionales. The phylogeny of selected proteins of the DNA maintenance program, and in particular those important for the replication/segregation of Chr.2 in *V. cholerae*, like those of the *dam*-cohort (Brézellec et al. 2006), supports this conclusion (supplementary table 2, Supplementary Material online). Our phylogenetic analysis shows that *P. shigelloides* split early in the branch of Enterobacterales, which emerged together with the branch of Vibrionales from the LCA of the whole clade. Several markers support this conclusion. The acquisition of *secM* and the absence of a *parAB* operon on the main chromosome of *P. shigelloides* are characteristic of the genomes of Enterobacterales. The absence of a genomic Ter/*tus* system (Galli et al. 2019), the persistence of the γ-flagellar system (Ferreira et al. 2021), and the arrangement of the *ori* region of the main chromosome of *P. shigelloides* (supplementary fig. 3, Supplementary Material online) substantiate an early speciation of *P. shigelloides* within the branch of Enterobacterales (inset in fig. 2). Altogether these results suggest that the most parsimonious hypothesis is that *P. shigelloides* is not a Vibrionales but an Enterobacterales.

We aimed to delineate phylogenetically the group of *Plesiomonas*-related species carrying a chromid to investigate the history of this branch of Enterobacterales. Yet, the closest species identified were all well-known monochromosome-Enterobacterales. We investigated the possibility that the genus *Plesiomonas* regrouped various species and concluded that the branch of *P. shigelloides* carries only a single species (supplementary material and fig. 4, Supplementary Material online). Hence, *P.shigelloides* is an Enterobacterales that carries within its genome a chromid whose replication initiation machinery is related to that of the Vibrionales. This situation is therefore particularly suitable to pursue our comparative study on the adaptive features needed to ensure the persistence of a chromid without interfering with the progression of the host cell cycle in *Vibrio* and in *Plesiomonas*.

### The *Plesiomonas* and *Vibrio* Chromids Carry Common Replication Regulation Features

**Fig. 3:**
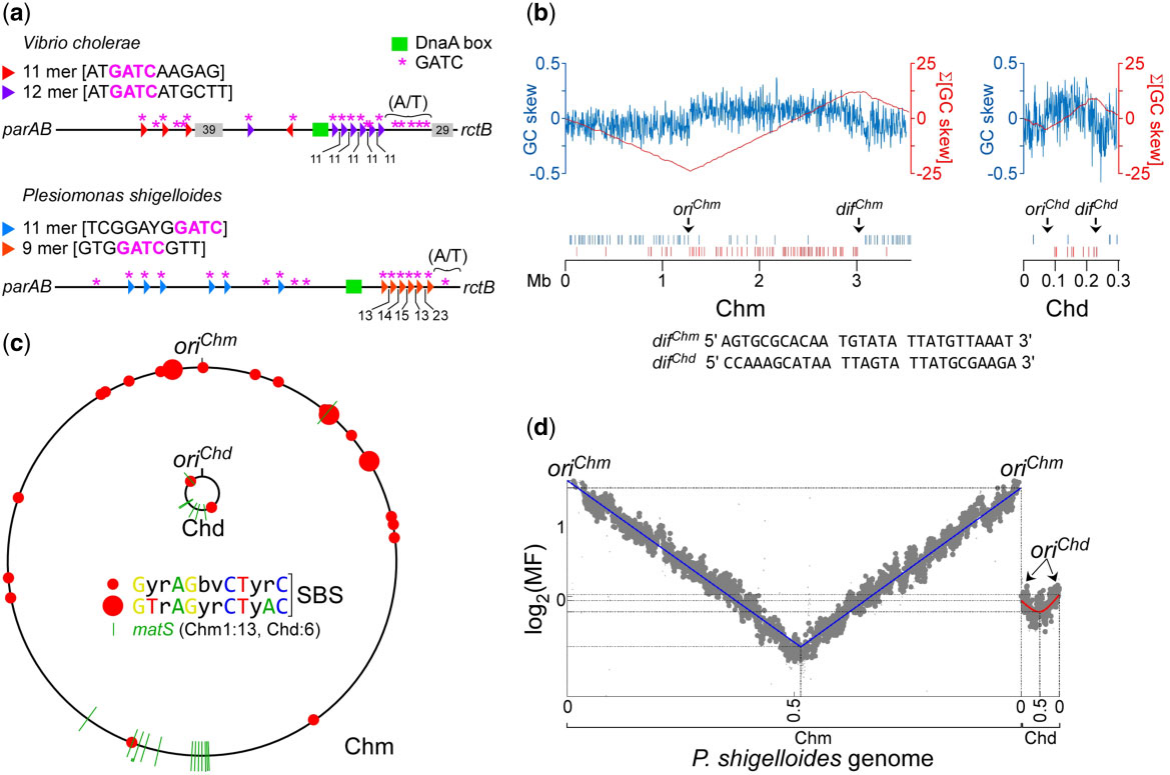
Organizational features of the genome of *P. shigelloides*. (*a*) Organization of the presumptive origin of replication of the replicon of *P. shigelloides*. The presumptive origin of replication of the replicon of *P. shigelloides*, spanning the noncoding sequence from *rctB* to *parAB* is displayed with salient features: GATC sites (*), iterons (colored arrowheads; the sequence of the two types of iterons is provided), DnaA box (green square). The A/T rich region is shown. (*b*) Calculation of the GC skew and cumulative GC skew on the main chromosome (3,555 kb) and the chromid (299 kb) and distribution of the KOPS on each replichore of each replicon. Each vertical bar represents an occurrence detected on the (+) strand (blue) and the (−) strand (red). Origin of replication on the bacterial chromosome (*ori^Chm^*) and upstream of the *rctB* gene on the chromid (*ori^Chd^*) are identified. The *dif* sites located on the bacterial chromosome (*dif^Chm^*) and on the replicon (*dif^Chd^*) are also identified. The sequences of *dif^Chm^* and *dif^Chd^* are provided. (*c*) Distribution of the SBS and *matS* on the two replicons. (*d*) Marker frequency analysis of *P. shigelloides* replication program. The analysis performed on DNA of exponentially growing cells follows the replication of the main chromosome (Chm) and of the RctB-chromid (Chd).

The presence of GATC sites in the 8 and the 10 mers iteron-like sequences within *ori*^Chd^ of *P. shigelloides* is strikingly reminiscent of the organization of *ori2* of *V. cholerae* (fig. 3*a*). It suggests that the affinity of RctB for the iterons at *ori*^Chd^ might be modulated by the methylation status of the origin of replication, in a way similar to that described in *V. cholerae*. It suggests that *dam*-dependent activities might be involved in the initiation control of the *P. shigelloides* chromid replication.

Another feature of the replication program in Vibrionales is that chromid replication occurs during that of Chr1. The coordination is such that the termination of the replication of the two chromosomes are synchronized, suggesting that it might have been an adaptation of the replication program of the chromid to the bacterium cell cycle. We performed a marker frequency analysis in *P. shigelloides* to investigate the timing of the replication of the chromid with respect to that of the main chromosome. We analyzed the nine strains of *P. shigelloides* described earlier (Materials and Methods). The DNA analyzed was extracted from exponentially growing cells and subjected to deep sequencing (Materials and Methods). Then, we used the genome of strain 7A as a matrix for the MF analysis of the eight other strains (Materials and Methods). Except in the strain NRC32124, the replication of the chromid occurred during that of the main chromosome, indicating that the completion of the replication of the main chromosome is not synchronized with that of the chromid in all the strains analyzed (fig. 3*d*; supplementary fig. 5, Supplementary Material online). The MF profile from NRC32124 cells is different from those obtained with the other strains, suggesting that the replication of the chromid occurred after completion of the replication of the main chromosome. Alternatively, it could indicate that the chromid was either lost or not replicated in a fraction of the cultivated cells. Yet, we could not purify chromid-less cells, suggesting that chromid-less cells are unviable.

### The *Plesiomonas* and *Vibrio* Chromids Carry Common Segregation Features

We searched the genome of *P. shigelloides* for other markers that might be important for the adaptation of the chromid to the cell cycle. Chromosome dimers occur during replication in a RecA-dependent manner. We searched the presence of *dif* sites in the replication fork-convergence region of the two replicons (fig. 3*b*). We identified a *dif* site on each replicon: A canonical site (*dif*^Chm^) was found in the terminus region of the main chromosome and a more degenerated site (*dif*^Chd^) was identified in the terminus region of the chromid (fig. 3*b*). In support, we identified numerous polarized KOPS on the bacterial chromosome and on the chromid pointing toward *dif*^Chm^ and *dif*^Chd^, suggesting that chromosome dimer resolution on both replicons is FtsK/XerCD-dependent (fig. 3*b*). Also, we identified numerous SlmA-binding sites (SBS) around *ori*^Chm^ suggesting an active nucleoid occlusion program in *P. shigelloides*. The situation was much less clear with respect to the presence of SBS on the chromid, likely as a consequence of the small size of the chromid (fig. 3*c*). Finally, we found that the terminus region of both chromosomes of *P. shigelloides* are both enriched in MatP-binding sites (fig. 3*c*). Altogether, these elements strongly suggest a long-standing relationship between the RctB-replicon and *P. shigelloides* and an advanced adaptation of the chromid to the cell cycle of *P. shigelloides*.

### DNA Maintenance Proteins of *Vibrio* and *Plesiomonas* Share Common Protein Signatures

Due to the integration of a chromid in the cell cycle of *P. shigelloides*, some DNA maintenance genes might have become essential, others might have acquired adaptive mutations.

**Fig. 4:**
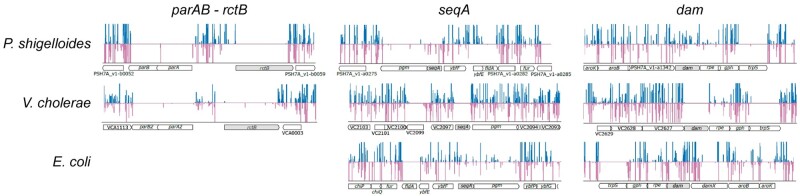
The chromid of *P. shigelloides* is essential for cell viability. Comparison of transposon insertion profiles of *P. shigelloides*, *V. cholerae*, and *E. coli* at *rctB*, *parAB*, *seqA* and *dam* genes (supplementary table 4, Supplementary Material online). The amplitude of the signal represents the relative insertions rate of Transposon at a given position (insertion at a given site/total number of reads). Data from *P. shigelloides* and *V. cholerae* correspond to the combination of three independent experiments. Those of *E. coli* correspond to a single Tn-seq experiment. Blue and pink bars indicate insertions in the forward and reverse strand, respectively.

We addressed the first question by determining the genes that could, or could not, be interrupted by Transposons in a Tn-seq experiment (Materials and Methods). We did not detect any Tn insertion in *rctB* and in the chromid *parAB* genes, indicating that the chromid is essential for the viability of *Plesiomonas* (fig. 4). *seqA* and *dam* were shown to be essential in *V. cholera*e but not in the monochromosomal bacteria *E. coli*. It is supposedly due to the implication of the gene products in the replication of Chr2. In contrast, Tn-seq data indicated that *seqA* and *dam* are facultative in *P. shigelloides*. Yet, the Tn integration frequency in *dam* was significantly lower than that expected for a nonessential gene, suggesting that the fitness of a *dam* null-mutant was reduced in *P. shigelloides* (fig. 4).

We initiated a comparative analysis of the sequence of various proteins of the DNA maintenance program of *P. shigelloides*, Vibrionales, and Enterobacterales. In particular, we searched *P. shigelloides* proteins for motifs that are typically present in Vibrionales and not in Enterobacterales proteins. The presence of *matS* sites in *ter*^Chd^, the numerous GATC sites within *ori*^Chd^ together with the reduced fitness of the *dam* mutant led us to focus on the genes of the *dam*-cohort (Brézellec et al. 2006).

**Fig. 5:**
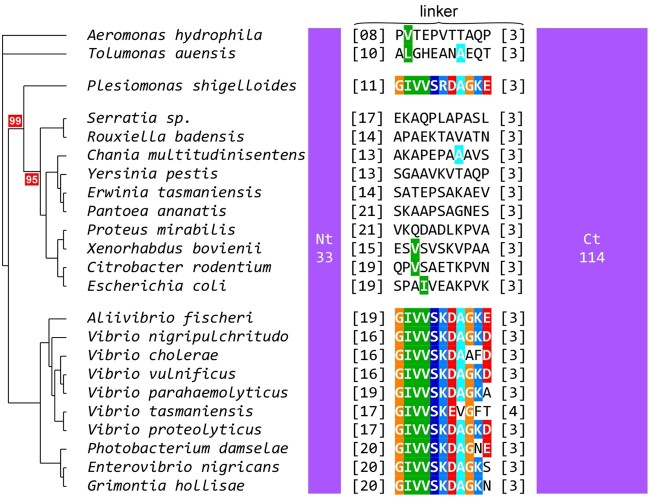
The linker domain of *Ps*SeqA possesses a signature typical of the Vibrionales. Phylogenetic tree based on the SeqA protein of representatives of the Enterobacterales, the Vibrioanales and the Aeromonadales. The NTD and CTD are represented as purple boxes. The size of each domain is indicated. The highly conserved sequence within the linker domain of Vibrionales as well as the conserved residues in the other sequences are highlighted. Red: acid; blue: basic; green: neutral. Relevant bootstrap scores are boxed in red.

**Fig. 6:**
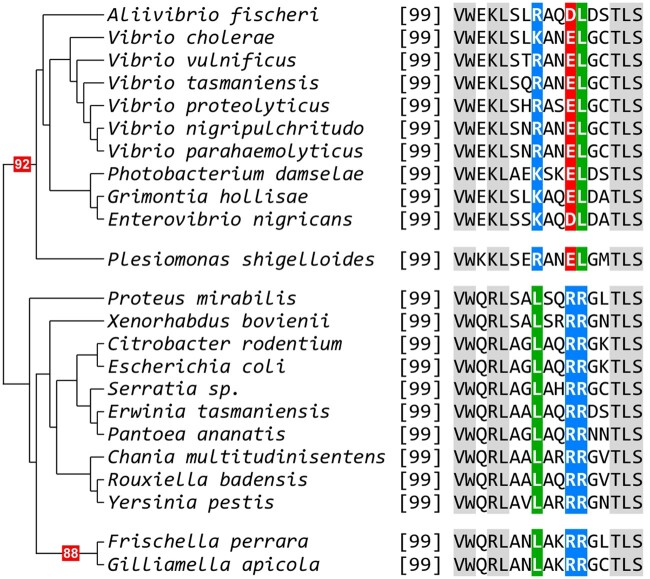
The dimerization domain of *Ps*MatP possesses a signature typical of the Vibrionales. Phylogenetic tree based on the MatP protein of representatives of the Enterobacterales and the Vibrioanales. The conserved motif found in Vibrionales and in *P. shigelloides* is highlighted. Conserved aminoacids are boxed in gray. Relevant bootstrap scores are boxed in red.

We detected a Vibrionales pattern in two proteins of *P. shigelloides*: *Ps*SeqA and *Ps*MatP. The alignment of SeqA proteins of Enterobacterales and Vibrionales revealed the strict conservation of a stretch of 11 amino acid long in Vibrionales—but not in Enterobacterales proteins, within the linker (connecting the NTD and the CTD of the protein) that was shown to be critical for the formation of oligomers of SeqA (Chung et al. 2009). Strikingly, this pattern was also found in *Ps*SeqA, although the protein is clearly phylogenetically rooted to the Enterobacterales clade (fig. 5). Similarly, *Ps*MatP carries a protein motif diagnostic of the MatP proteins of Vibrionales, which is located within the MatP dimerization domain (fig. 6) (Dupaigne et al. 2012).

### Order-Specific Domains Enriched in Ecological Niche-Relevant Activities

The presence of a *rctB*-chromid in *P. shigelloides* led us to consider that the LCA of Enterobacterales and Vibrionales might have already carried a *rctB*-chromid. According to this scenario, the nascent group of Enterobacterales split early in two sub-branches. One sub-branch lost secondarily the *rctB*-chromid to give the clade regrouping bona fide Enterobacterales, whereas the other sub-branch gave *P. shigelloides*. Hence, we hypothesized that *P. shigelloides* might have retained through evolution activities that are important for the maintenance of the chromid and that might have been lost by lack of pressure of selection in the bona fide Enterobacterales, that is, in Enterobacterales that lost the chromid. To investigate this hypothesis and identify these activities, we developed a global approach based on the analysis of the distribution of Pfam domains in these species.

Pfam domains are autonomous structural and/or functional elements of proteins defined by their HMM signature. Conveniently, their signature persists through evolution almost independently of their degree of relatedness. Proteins may contain several domains and each domain may be present in several proteins, making the distribution of domains within a proteome a handy marker of its history. We defined the “domainome” of an individual as the set of domains present in its proteome and by analogy with genomic cladistics, we defined “pan-domainome” and “core-domainome” as the set of domains found in the proteome of at least one and of all individuals in a given clade, respectively. Because domains are acquired and/or lost through vertical and/or horizontal transfer, the core-domainome regroups the domains that are expected to be under pressure of selection in the species composing the clade. Conversely, the domains of the core-domainome of a given clade that are systematically absent from the pan-domainome of a different but closely related group of species (i.e., with a common ancestor) are likely to be clade specific features.

**Fig. 7:**
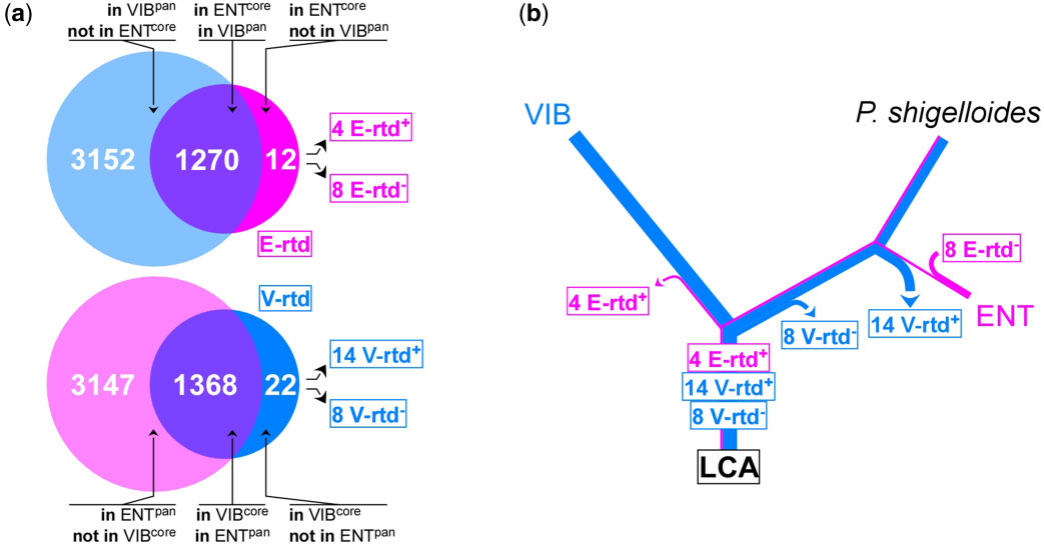
*Plesiomonas shigelloides* possesses Vibrionales- and Enterobacterales-restricted domains. (*a*) The core/pan-domainome of the Enterobacterales and Vibrionales were built to identify the Enterobacterales- and the Vibrionales-restricted domains. Thirteen Enterobacterales (E-rtd) and 22 Vibrionales domains (V-rtd) were identified as “restricted” to each order, respectively. Among these domains, some were found in *P. shigelloides* (E-rtd^+^ and V-rtd^+^) and some were not (E-rtd^−^ and V-rtd^−^). (*b*) Plausible history of the gains and losses of E-rtd and V-rtd domains through evolution since the LCA. Stroke thickness is an arithmetic function of the number of domains.

We established the core- and the pan-domainomes of the Enterobacterales and the Vibrionales out of 21 proteomes of each order (fig. 7*a*; supplementary table 1, Supplementary Material online). The choice of these proteomes was based on the representativity of the species picked and on the completeness of their genome assembly (supplementary table 1, Supplementary Material online). The pan-domainome of the Vibrionales (VIB^pan^) and Enterobacterales (ENT^pan^) regroups 4,422 and 4,515 domains, respectively, whereas the core-domainome of the same orders contains 1,282 (ENT^core^) and 1,389 (VIB^core^) domains. In total, we identified 12 and 22 order-restricted domains within the core-domainome of the Enterobacterales (E-rtd) and the Vibrionales (V-rtd), respectively (fig. 7*a*).

The distribution of the V-rtd domains contrasts with that of the E-rtd domains. Most E-rtd (8 out of 12) are strictly restricted to this order. None of them was found in *P. shigelloides*, suggesting that these domains were acquired recently (fig. 7*b*). In comparison, and except for the two domains present in RctB (HTH_55 and DUF3346; supplementary table 1, Supplementary Material online), all V-rtd domains are broadly distributed among bacteria, archeae and eukaryotes and 14 out of 22 were identified in *P. shigelloides* (fig. 7*b*; supplementary table 1, Supplementary Material online).

Altogether, these results reveal two distinct trends. V-rtd domains were likely present in the LCA, maintained under pressure of selection in Vibrionales and lost by waves in the branch leading to the Enterobacterales: Eight were lost early after the isolation of the Enterobacterales branch along with the main chromosome *parAB* operon (fig. 7*b*, inset in fig. 2) and 14 were lost after the separation from the *P. shigelloides* branch (fig. 7*b*). In contrast, the majority of the Enterobacterales-restricted domains (8 out of 12) were acquired recently in the Enterobacterales, after the separation from the *P. shigelloides* branch (fig. 7*b*).

## Discussion

In this report, we established that a chromid related to the Chr2 of Vibrionales belongs to the genome of *P.shigelloides*. We demonstrated that *P. shigelloides* is an atypical Enterobacterales through phylogenetic analyses based on various molecular markers, genomic reorganization scars and gene loss or acquisition. This led us to initiate a comparative analysis to evaluate the relevance and the ubiquity of the various genetic traits that were proposed to contribute to the maintenance of chromids in Vibrionales.

Several genomic structural elements typically identified on the main chromosome were also detected on the chromid, suggesting that they are a requirement for their maintenance. Numerous KOPS were found on each replichore and the presence of a *dif* sequence strongly suggests that chromid-dimers in *P. shigelloides*, like in *V. cholerae*, are resolved through the XerCD recombinase system. Also, the large number of *matS* sites within the replication fork convergence region suggests that the newly replicated copies of the terminus region of the *P. shigelloides* chromid are positioned at midcell through the *matS*/MatP system. We found, however, only two SBS motifs on the chromid, suggesting that it is unlikely to contribute to the proper licensing of cell division by nucleoid occlusion. We showed that the termination of the replication of the chromid is not synchronized with that of the bacterial chromosome and furthermore that the timing of replication initiation varied from strain to strain. Nevertheless, the copy number of the RctB-chromid was modeled on that of the main chromosome (supplementary fig. 5, Supplementary Material online). It is too early, however, to conclude that synchronization of the termination of replications is not an important feature of chromids. As mentioned earlier, we observed a deficit of RctB-chromid replication in cultivated NRC31124 cells. We identified a strain of *Plesiomonas shigelloides* without RctB-chromid (NCTC10360). We resequenced this strain to confirm that no part of the chromid was integrated on the main chromosome. We observed that the growth of these two strains (NRC31124 and NCTC10360) was extremely poor (data not shown) and no chromid-less NRC31124 cells could be purified. This indicates that chromid loss seriously and durably compromises the fitness of *P. shigelloides*. Alternatively, it may reflect the fact that the replication of the chromid is still not completely integrated within the host cell cycle.

We identified two genetic signatures that might reveal cellular adaptations to the integration of the replication/segregation of the chromid in the host cell cycle. The first was identified in SeqA. We noticed a protein motif in the linker of *Ps*SeqA similar to that found in Vibrionales. The function of the linker was associated with the flexibility of SeqA in *E. coli*. Active SeqA exist as dimers, in which the NTDs ensures the interaction between two subunits whereas the CTDs interacts with two distinct hemimethylated GATC sites. EMSA experiments revealed that the linker enables long distance interactions between dimers of SeqA and two hemimethylated GATC sites (Guarné et al. 2005). In Enterobacterales, the linker is unstructured and its sequence is random, consistent with its role in the plasticity of the protein. In contrast, the linker found in the SeqA proteins of Vibrionales and *P. shigelloides*, contains a highly conserved protein motif, indicating that a strong pressure of selection is applied on this sequence in this group of species. It would be interesting to investigate whether the linker of SeqA in Vibrionales and in *P. shigelloides* specifies an additional function in connection with the maintenance of the RctB-chromid. The second genetic signature typical of the Vibrionales that was found in a *P. shigelloides* protein is located within ZapB-binding domain of MatP, at the Carboxy terminal end of helix α5 (Dupaigne et al. 2012). Indeed, *Ps*MatP falls cladistically within the group of the Vibrionales. In *E. coli* and on Chr1 of *V. cholerae*, the segregation of the Ter macrodomain is delayed through a MatP-dependent interaction with the divisome at midcell. Although MatP appears to be also involved in the positioning of the chromid of *V. cholerae*, the connection between the two newly replicated chromid Ter regions was shown to be looser, suggesting an adaptation of MatP to the segregation of two types of replicon (Demarre et al. 2014). Given the constraint similarities in term of genome maintenance between *V. cholerae* and *P. shigelloides*, it would be interesting to investigate whether the common genetic signature detected within the dimerization domain of MatP could be an adaptation to the segregation of the two replicons.

The domainome approach aimed at identifying relevant activities with respect to the maintenance of RctB-chromids led to the identification of two domains of unknown function: PF12069 and PF12119. The proteins associated with these two domains are present in the proteome of all Vibrionales investigated and in *P. shigelloides* and define a monophyletic group (supplementary fig. 6*a* and *b*, Supplementary Material online). The PF12119-containing gene is especially interesting because it is distributed typically like a mobile element outside the Vibrionales/*Plesiomonas* group, suggesting that the gene is under pressure of selection and inherited vertically in the Vibrionales/*Plesiomonas* group. It could be linked to an implication on the maintenance of the RctB-chromid. Alternatively, the acquisition of these genes could result from the adaptability of the bacterial genomes to their environment. Indeed, six Vibrionales-restricted domains found in *P. shigelloides* are protein components of the polar flagellum. The H- and T-ring contribute to the formation of a periplasmic basal disk associated with flagellar motor, whereas MotY, FlgO, and FlgT assist the assembly of the flagellum (Beeby et al. 2016). Polar flagellum systems are found in several orders of the marine γ-proteobacteria (Vibrionales, Aeromonadales, Alteromonadales, or Pseudomonadales). This type of flagellum develops a motor of higher torque than that found in *E. coli* and *Salmonella typhimurium*, which appears to be particularly adapted for fast movements in an aquatic environment. Enterobacterales—like *E.coli—*on the other hand lost the γ-proteobacterial polar motor during evolution, probably while adapting to their enteric niche. They acquired through horizontal transfer a more suitable β-proteobacterial flagellar system (Ferreira et al. 2021).

### Concluding Remarks

It is difficult to perceive the immediate interest for the cell to ensure the maintenance of an additional essential replicon, especially if its proper maintenance requires the adaptation of replication-related genes. Several hypotheses were proposed to justify the advantage for a cell to have a multipartite genome like extending the size of the genome, or accelerating the growth rate or even helping the cell adapt to new biological niches. It was also suggested that chromids might be an interesting mean to modulate protein expression by gene dosage. Yet, several counter examples challenge each of these hypotheses (diCenzo and Finan 2017), not mentioning that the high percentage of bacterial species, whose genome is contained on a single chromosome (∼90%) rather suggests that multipartite genomes are accidental situations, imposed and enforced by the plasmid through toxin/antitoxin genes, for example. The strategies aimed at integrating the replication of a chromid within the host cell cycle might simply seek to perturb it as minimally as possible.

## Materials and Methods

### Strains, Media, and Growth

Nine strains of *Plesiomonas shigelloides* were analyzed in this study (the place of sampling is indicated under bracket). NRC32124 (river, Czeck republic), O164 (fish, sweden), NRC2599 (lake, Slovakia), 8A (lake, Sweden), 7A (river, Sweden), NRC73297 (river, Slovakia), NRC89372 (sewage, Slovakia), NRC88238 (lake, Slovakia), and RS29480 (human, Slovakia). Strain of *V.cholerae* used for the Tn-seq. For Tn-seq analysis *E. coli* β2163 + pEE18 was used as donor strain. The strains of *P.shigelloides* used in this study were grown in LB at 25 °C. When needed, media was supplemented with Cloramphenicol (25 µg/ml), Kanamycin (50 µg/ml), and d-aminopimelic acid (0.3 mM). Solid media contained 1.5% agarose.

### Domainome

#### Data Sets

Genomes of Enterobacterales used in this study: *Citrobacter koseri* (strain ATCC BAA-895/CDC 4225-83/SGSC4696), *Cedecea davisae* DSM 4568, *Klebsiella pneumoniae* subsp. *pneumoniae* (strain ATCC 700721/MGH 78578), *E.coli* (strain K12), *Erwinia tasmaniensis* (strain DSM 17950/CIP 109463/Et1/99), *Tatumella ptyseos* ATCC 33301, *Pantoea ananatis* (strain LMG 20103), *Pectobacterium atrosepticum* (strain SCRI 1043/ATCC BAA-672), *Erwinia carotovora* subsp. *atroseptica*, *Brenneria goodwinii*, *Dickeya dadantii* (strain 3937), *Erwinia chrysanthemi* (strain 3937), *Lonsdalea quercina*, *Yersinia pestis*, *Serratia fonticola* AU-P3(3), *Rahnella aquatilis* (strain ATCC 33071/DSM 4594/JCM 1683/NBRC 105701/NCIMB 13365/CIP 78.65), *Hafnia alvei* FB1, *Edwardsiella ictaluri* (strain 93-146), *Morganella morganii* subsp. *morganii* KT, *Photorhabdus luminescens* subsp. *laumondii* (strain DSM 15139/CIP 105565/TT01), *Xenorhabdus bovienii* (strain SS-2004), *Pragia fontium*, *Leminorella grimontii* ATCC 33999 = DSM 5078, *Plesiomonas shigelloides* 302-73, *Plesiomonas shigelloides* 7a. Genomes of Vibrionales used in this study: *Aliivibrio fischeri* (strain ATCC 700601/ES114), *Vibrio fischeri*, *Grimontia hollisae* CIP 101886, *Photobacterium damselae* subsp. *damselae* CIP 102761, *Photobacterium profundum* (strain SS9), *Enterovibrio nigricans* DSM 22720, *Vibrio aerogenes* CECT 7868, *V.cholerae* serotype O1 (strain ATCC 39315/El Tor Inaba N16961), *Vibrio azureus* NBRC 104587, *Vibrio furnissii* (strain DSM 14383/NCTC 11218/VL 6966), *Vibrio nigripulchritudo*, *Vibrio parahaemolyticus* serotype O3: K6 (strain RIMD 2210633), *Vibrio proteolyticus* NBRC 13287, *Vibrio tasmaniensis* (strain LGP32), *Vibrio splendidus* (strain Mel32), *Vibrio vulnificus* (strain YJ016), *Vibrio xiamenensis*, *Vibrio variabilis*, *Vibrio caribbeanicus* ATCC BAA-2122, *Vibrio metschnikovii* CIP 69.14, *Vibrio diazotrophicus*, *Vibrio nereis*, and *Vibrio palustris*.

#### Pfam Annotations

The Pfam annotations pertaining to the above-mentioned bacteria were downloaded from the Pfam site (http://pfam.xfam.org/) release 34.0.

#### Computation of “Order-Specific Domains”

Order-specific domains were obtained using “DomainSieve,” a soft we developed in 2006 (Brézellec et al. 2006). “DomainSieve” works with Pfam protein domains. It collects protein domains that are systematically present in a set of “in-group” organisms and excludes those that are also identified in at least one organism of the “out-group” set of organisms. Thus, for instance, the computation of E-rtd domains is obtained using the intersection of the set of Enterobacterales organisms as “in-group” and the union of the set of Vibrional organisms as “out-group.”

### Phylogenetic Analyses

An alignment of proteins (a crude alignment generated using the program ClustalW and refined by hand) was fueled into PhyLM (v.3.0) and 100 bootstrap replicates were generated for each analysis (Guindon and Gascuel 2003). A consensus tree was eventually obtained by running the program CONSENSE and fed as an input tree into PhyML. Significant bootstrap scores (arbitrarily above 70%) are indicated.

### Marker Frequency Analysis

Overnight cultures of *P. shigelloides* were diluted in 10 ml of fresh media to a final OD_600_ 0.01. Cells were grown until they reached an OD_600_ 0.1 and rediluted in 10 ml of fresh media to a final OD_600_ 0.01. Once cultures reached OD_600_ 0.1, cells were collected and subjected to genomic DNA extraction. Extraction of gDNA was performed using the Sigma GenElute bacterial genomic DNA kit and sequencing libraries were prepared using the NEBNext Ultra II FS DNA Library Prep Kit for Illumina kit. Paired-end sequencing was performed in a NextSeq (Illumina). Number of reads used for the MFA of each strain analyzed: PSH1 (34629688), PSH2 (31705987), PSH3 (27088120), PSH4 (36719732), PSH5 (42314957), PSH6 (28884789), PSH7 (31947357), PSH8 (29756969), and PSH9 (33285060). Sequence data analysis was performed as described in Galli et al. (2019) with small modifications. Reads of all *P. shigelloides* strains were aligned on *P. shigelloides* PSH5 genome and sequences not aligned were filtered out. Enriched regions were calculated using a 2- and 500-kb window, values from 2-kb window deviating more than 16% from 500-kb window were discarded.

### Tn-Seq Analysis

Samples for Tn-seq analysis were prepared as described (Espinosa et al. 2020). Libraries were paired-end sequenced in a Next-Seq (Illumina). Sequencing data analysis was performed as described (Espinosa et al. 2020). Transposon insertions at *dam*, *seqA*, and *rctB* genes were visualized using ARTEMIS (Carver et al. 2012).

### Sequencing

Genomic DNA extracted from *E. coli* was used for Illumina or Oxford nanopore Technologies (ONT) sequencing library preparation. Illumina libraries were prepared following the standard TruSeq protocol with mechanical shearing (Covaris S220), end-repair, A-tailing and adapter ligation followed by PCR amplification. Illumina libraries were sequenced on a NextSeq500 platform (paired end 2 × 75 bp). For nanopore sequencing library preparation the SQK-NSK007 kit from ONT was used with the corresponding protocol. DNA was sheared using Covaris g-TUBEs to generate approximately 8 kb fragments followed by the optional DNA repair step, end-repair, and adapter ligation. The resulting library was loaded onto a MinION flow cell version 7.3 and sequenced on a MinION Mk1B sequencer. The ONT run generated 28 194 “2D” reads (∼137 Mb). Base calling was performed using the Oxford Nanopore base callers (Metrichor). Canu was used for correction, trimming, and assembly of reads to produce one assembly of three contigs (3,555,866, 298,933, and 5,987 pb) (Koren et al. 2017). Pilon was used for assembly polishing with the Illumina sequences described above. We performed six rounds with “bases” mode correction and one final round with “all” mode correction (Walker et al. 2014). 

### *Plesiomonas shigelloides* 7A Reannotation

The main annotations of *P. shigelloides* 7A was performed on the MicroScope annotation platform. For proteins annotated as “unknown function” by Microscope, we triggered a reannotation pipeline in order to attribute, when available, Pfam domains to these proteins. To achieve this goal, the HHsuite package was used as follows:

1. Using HHBlits, the primary sequence of each protein annotated as “unknown” was ran against the UniRef30 database in order to create a multiple sequence alignment (MSA).
2. An HMM profile was then then created from this MSA.
3. To improve the robustness of the profile, the secondary structure prediction of the unknown protein was integrated to the HMM.
4. The profile obtained was compared with the Pfam database (32.0) with HHSearch.
5. We wrote a python script to collect the protein sequences in which we identified Pfam domains whose probability to be present in the given protein was above 99.00%. When more than one such domain were identified in the same protein sequence, the domain with the highest score was retained.

## Supplementary Material

Supplementary data are available at *Genome Biology and Evolution* online.

## Supplementary Material

evac011_Supplementary_Data
