# Supplementary material for: Plesiomonas shigelloides, an Atypical Enterobacterales with a Vibrio-Related Secondary Chromosome
Source: Genome Biol Evol. 2022 Jan 25;14(2):evac011. doi: 10.1093/gbe/evac011 (PMC8826520; doi:10.1093/gbe/evac011)
Supplement: evac011_Supplementary_Data [file evac011_supplementary_data.zip › Plesio_paper_sup_Figures_legends.docx]

**Supplementary figure legends**

**Supplementary fig. 1. RctB contains a distinctive protein domain.** RctB is related to various plasmid replication initiators (RepA, TrfA, RepC) suggesting that they all derive from a common ancestor. Yet, domain PF11828 (DUF3346) distinguish RctB from the other replication initiator proteins

**Supplementary fig. 2.** **Organization of the presumptive origin of replication of the chromid of *P. shigelloides* (*ori^Chd^*).** The position of *parA* and *rctB* (boxed in blue) are indicated. The 9 sequences of the species that were used in this study together with the 4 sequences available at uniprot were aligned, revealing a conserved and unique DnaA binding site (shaded in green). Two repeated sequences (iteron-like) were identified: six 8 mers sequences from the DnaA binding site to *rctB* and six 10 mers sequences form the DnaA binding site to *parA*. (bottom) Surrounding sequences of the two types of iteron were aligned to determine the exact size of the repeated sequences

**Supplementary fig. 3. Rearrangement of the chromosome origin region within the Enterobacterales.** The genes present within the genome region from *dnaA* to *mioC* are represented in colored blocks. The *dnaA* block (light red) contain the genes from *dnaA* to *trmE*. This block is well conserved and the genes are collinear among all the species analyzed. The *atp* block (light purple) contain the *atp* operon and the *oriC* block (light green) contains the genes from *mioC* to *gidB*. A rearrangement of the region containing the *atp* and the *oriC* blocks occurred in the Enterobacterales after the split of *Plesiomonas shigelloides* from the main branch of the Enterobacterales. Other markers (loss of *parAB*, the gain of *tus* and the replacement of *dciA* by *dnaC*) are indicated.

**Supplementary fig. 4. Plesiomonas shigelloides is an orphan Enterobacterales.** Salerno and coll., reported a high nucleotide diversity within the species after analyzing the sequence of amplification products corresponding to sections of housekeeping genes (Salerno et al, 2007). We therefore investigated the possibility that *P. shigelloides* be in fact a genus and not a species. To decipher the taxonomic status of *P. shigelloides*, we extended the analysis to 6 single housekeeping genes involved in various biological functions (DNA repair, chromosome organization, transcription, metabolism), representing a total of > 22 kb of DNA and > 7500 aa (supplementary Table 3). The analysis was carried out in *P. shigelloides* as well as in two species (whose genomic sequences are similarly abundant in the databank) of the Enterobacterales (*Citrobacter* *Koseri*) and the Vibrionales (*Photobacterium damselae*). We also performed the same analysis in the genus *Citrobacter*. **(a)** Cladogram of the Enterobacterales. *Photobacterium damselae* is used as an outgroup. Strains used to calculate DNA and protein distances within a species are indicated by a colored triangle. Species used to calculate DNA and protein distances within a genus are boxed in green. **(b)** DNA and protein distances were calculated according to the F84 and the Jones-Taylor-Thronton models, respectively, on 6 single and housekeeping genes (see sup_table 3). The distribution of the medians is represented for 2 species (*Citrobacter koseri* and *Photobacterium damselae*) one genus (*Citrobacter*) and *P. shigelloides*. The box plot display the lower and upper adjacent values, the upper and lower quartile (rectangle), the median (horizontal bar) and the average (cross). The close dot points to the median distance obtained for RctB in *P. shigelloides*. The nucleotide diversity in *P. shigelloides* is 2 to 3 times more important than in *C. koseri* or *P.* *damselae*, but between 5 and 10 times lower than in the genus *Citrobacter*. We conclude that *P. shigelloides* is an orphan species within the order Enterobacterales.

**Supplementary fig. 5. Marker frequency analysis of *P. shigelloides*.** Marker frequency analysis of *P. shigelloides* strains used in this study. Marker frequencies are represented in log2. The section corresponding to the Chromosome (Chm) and the Chromid (Chd) are indicated at the bottom of the graph, together with representative features (*ori* and *dif*) on each replicon. Fitting curves representating the evolution of the marker frequencies along each replicon (blue for the chromosome and red for the chromid) are also provided.

**Supplementary fig. 6. Phylogenetic tree of two V-rtd domains of *P. shigelloides*.** The phylogeny of PF12069- and PF12119-containing proteins is presented. Relevant bootstrap scores and a scale indicative of the substitution frequency are provided.
