# Supplementary material for: Plesiomonas shigelloides, an Atypical Enterobacterales with a Vibrio-Related Secondary Chromosome
Source: Genome Biol Evol. 2022 Jan 25;14(2):evac011. doi: 10.1093/gbe/evac011 (PMC8826520; doi:10.1093/gbe/evac011)
Supplement: evac011_Supplementary_Data [file evac011_supplementary_data.zip › sup_Figure 2.docx]

NRC32124 CATCGCCTTCCTCGTCAAAGTGTCATCCTCTTTAAT-AAGAATAATGATT

*parA*

O164 CATCGCCTTCCTCGTCAAAGTGTCATCCTCTTTAATTAAGGATAATGATT

NRC2599 CATCGCCTTCCTCGTCAAAGTGTCATCCTCTTTAAT-AAGGATAATGATT

8A CATCGCCTTCCTCGTCAAAGTGTCATCCTCTTTAATTAAGGATAATGATT

7A CATCGCCTTCCTCGTCAAAGTGTCATCCTCTTTAAT-AAGGATAATGATT

NRC73297 CATCGCCTTCCTCGTCAAAGTGTCATCCTCTTTAATTAAGGATAATGATT

NRC89372 CATCGCCTTCCTCGTCAAAGTGTCATCCTCTTTAATTAAGGATAATGATT

NRC88238 CATCGCCTTCCTCGTCAAAGTATCATCCTCTTTAAT-AAGGATAATGATT

RS29480 CATCGCCTTCCTCGTCAAAGTGTCATCCTCTTTAATTAAGTATAATGATT

[302-73](https://www.uniprot.org/taxonomy/1315976) CATCGCCTTCCTCGTCAAAGTGTCATCCTCTTTAAT-AAGGATAATGATT

C7R88 CATCGCCTTCCTCGTCAAAGTGTCATCCTCTTTAAT-AAGGATAATGATT

NCTC10363 CATCGCCTTCCTCGTCAAAGTGTCATCCTCTTTAAT-AAGGATAATGATT

FOC33 CATCGCCTTCCTCGTCAAAGTGTCATCCTCTTTAAT-AAGGATAATGATT

ACAACACTTAAAGCACATTGCAACACTTTTGGATTATTTTATCGCATTTTTCAAAAATCACATCTGATATAGCCAAGCGTTCTATTTCCATCAAAGCAAG

ACAACACTTAAAGCACATTGCAACACTTTTGGATTATTTTATCGTGTTTTTCAAAAATCACGTCTGATATAGCCAAGCGTTCTATTTCCATCAAAGCAAG

ACAACACTTAAAGCACATTGCAACACTTTTGGATTATTTTATCGCATTTTTCAAAAATCACATCTGATATAGCCAAGCGTTCTATTTCCATCAAGGCAAG

ACAACACTTAAAGCACATTGCAACACTTTTGGATTATTTTATCGTGTTTTTCAAAAATCACGTCTGATATAGCCAAGCGTTCTATTTCCATCAAAGCAAG

ACAACACTTAAAGCACATTGCAACACTTTTGGATTATTTTAACGCATTTTTCAAAAATCACATCTGATATAGCCAAGCGTTCTATTTCCATCAAGGCAAG

ACAACACTTAAAGCACATTGCAACACTTTTGGATTATTTTATCGCATTTTTCAAAAATCACGTCTGATATAGCCAAGCGTTCTATTTCCATCAAAGCAAG

ACAACACTTAAAGCACATTGCAACACTTTTGGATTATTTTATCGCATTTTTCAAAAATCACATCTGATATAGCCAAGCGTTCTATTTCCATCAAGGCAAG

ACAACACTTAAAGCACATTGCAACACTTTTGGATTATTTTATCGCATTTTTCAAAAATCACATCTGATATAGCCAAGCGTTCTATTTCCATCAAAGCAAG

ACAACACTTAAAGCACATTGCAACACTTTTGGATTATTTTATCGTGTTTTTCAAAAATCACGTCTGATATAGCCGAGCGTTCTATTTCCATCAAAGCAAG

ACAACACTTAAAGCACATTGCAACACTTTTGGATTATTTTATCGCATTTTTCAAAAATCACATCTGATATAGCCAAGCGTTCTATTTCCATCAAAGCAAG

ACAACACTTAAAGCACATTGCAACACTTTTGGATTATTTTATCGTGTTTTTCAAAAATCACGTCTGATATAGCCAAGCGTTCTATTTCCATCAAGGCAAG

ACAACACTTAAAGCACATTGCAACACTTTTGGATTATTTTATCGTATTTTTCAAAAATCACATCTGATATAGCCAAGCGTTCTATTTCCATCAAAGCAAG

ACAACACTTAAAGCACATTGCAACACTTTTTGGTTATTTTATCGTTTTTTTCAAAAATCGCATCTGATATAGCCAAGCGTTCTATTTCCATCAAAGCAAG

GCAA------------------------------CCTACATTAGCAGCTTGATAACATCACTTAGCACGAACATCATCAACAAACAGCAAAAACACGCTA

GCAA------------------------------CCTACGTTAGCAGCTTGATAAGATCACTCCGTACGAACATCAGCAACAAACAACAAAAACACGCTA

GCAAGGCAAGGCAAGGCAA---------------CCTACATTAGCAGCTTGATAACATCACTTAGCACGAACATCATCAACAAACAGCAAAAACACGCTA

GCAAGGCAAGGCAAGGCAAGGCAAG-----GCAACCTACATTAGCAGCTTGATAACATCACTTAGCACGAACATCATCAACAAACAGCAAAG-CACGCTA

GCAAGGCAAGGCAAGGCAAGG----------CAACCTACATTAGCAGCTTGATAAGATCACTCCGTACGAACATCAGCAACAAACAACAAAAACACGCTA

GCAA------------------------------CCTACGTTAGCAGCTTGATAAGATCACTTAGCACGAACATCATCAACAAACAGCAAAG-CACGCTA

GCAAGGCAAGGCAAGGCAA---------------CCTACATTAGCAGCTTGATAACATCACTTAGCACGAACATCATCAACAAACAGCAAAG-CACGCTA

GCAA------------------------------CCTACATTAGCAGCTTGATAACATCACTTAGCACGAACATCATCAACAAACAGCAAAAACACGCTA

GCAA------------------------------CCTACGTTAGCAGCTTGATAAGATCACTCCATACGAACATCAGCAACAAACAACAAAAACACGCTA

GCAA------------------------------CCTACATTAGCAGCTTGATAAGATCACTTAGCACGAATATCAGCAACAAACAGCAAAAACACGCTA

GCAAGGCAAGGCAAGGCAAGGCAAGGCAAGGCAACCTACATTAGCAGCTTGATAAGATCACTTAGCACGAACATCATCAACAAACAGCAAAAACACGCTA

GCAAGGCAAGGCAAGGCAAGG----------CAACCCACATTAGCAGCTTGATAAGATCACTCCGTACGAACATCAGCAACAAACAACAAAAACACGCTA

GCAA------------------------------CCTACATTAGCAGCTTGATAACATCACTTAGCACGAACATCATCAACAAACAGCAAAAACACGCTA

AGCCGACACGGGGTTAATCGACTTCCGCCATCACCCATTCAGCCGGAATAGCCTCGGACGGATCGCAATCAAAATAGCCTCATCCACCAGTAAATCAGGC

AGCCGATACAGGGTTAATCGACTTCAGCCCCC-CCCATTCAGCCGGAATAGCCTCGGACGGATCGCAATCAAAATAGCCTCACCCACCAGCAAATCAGGC

AGCCGACACGGGGTTAATCGACTTCCGCCATCACCCACTCAGCCGGAACAGCCTCGGACGGATCGCAATCAAAATAGCCTCACCCACCAGTAAATCAGGC

AGCCGACAC-GAGTTAATCGACTTCCGCC--CCCCCATTCAGCCGGAACAGCCTCGGACGGATCGCAATCAAAATAGCCTCACTCACCAGCAAATCAGGC

AGCCGACACAGGGTTAATCGACTTCCGCCATCACCCATTCAGCCGGAACAGCCTCGGACGGATCGCAATCAAAATAGCCTCACCCACCATCAAATCAGGT

AGCCGACAC-GGGTTAATCGACTTTATCCATCGCCCATTCAGCCGGAACAGCCTCGGACGGATCGCAATCAAAATAGCCTCACTCACCAGCAAATCAGGC

AGCCGACAC-GGGTTAATCGACTTTCTCCATCGCCCATTCAGCCGGAACAGCCTCGGACGGATCGCAATCAAAATAGCCTCACTCACCAGCAAATCAGGC

AGCCGACACGGGGTTAATCGACTTCCGCCATCACCCACTCAGCCGGAACAGCCTCGGACGGATCGCAATCAAAATAGCCTCACCCACCAGTAAATCAGGC

AGCCGACACAGGGTTAATCGACTTCCGCC---CCCCATTCAGCCGGAATAGCCTCGGACGGATCGCAATCAAAATAGCCTCACCCACCAGCAAATCAGGC

AGCCGACACAGGGTTAATCGACTTCCGCCATCACCCACTCAGCCGGAACAGCCTCGGACGGATCGCAATCAAAATAGCCTCACCCACCAGCAAATCAGGC

AGCCGACACAGGGTTAATCGACTTCCGCC--CCCCCATTCATCCGGAATAGCCTCGGACGGATCGCAATCAAAATAGCCTCACCCACCAGCAAATCAGAC

AGCCGACACAGGGTTAATCGACTTCCGCCATCACCCATTCAGCCGGAACAGCCTCGGACGGATCGCAATCAAAATAGCCTCACCCACCAGCAAATCAGGC

AGCCGACAC-GGGTTAATCGACTTCCGCC--CCCCCATTCAGCCGGAACAGCCTCGGACGGATCGCAATCAAAATAGCCTCACTCACCAGCAAATCAGGC

GGAATCACTTCGGATGGATCATGATTAAATTGACTAACGTCAGCGAATGACAAAACCTGCGGAATACACTCGGATGGATCGAAATTAAACCCAGACACCC

GGAATTACTTCGGATGGATCATGATTAAATTGGCTAACGTCAACGAATGATAAAACCTGCGGAATATACTCGGATGGATCGAAGTTAAACCCAGCAATCC

GGAATCACTTCGGATGGATCATGATTAAATTGACTAACGTCAGCGAATGACAAAACCTGCGGAATACACTCGGATGGATCGAAATTAAACCCAGACACCC

GGAATCACTTCGGATGGATCATGATTAAATTAGCTAACGTCAGCGAATGATAAAACCTGCGGAATACACTCGGATGGATCGAAGTTAAACCCAGCCATCC

GGAATCACTTCGGATGGATCATGATTAAATTGGCTAACGTCAGCGAATGATAAAACCTGCGGAATGCACTCGGATGGATCGAGGTTAAACCCAGCCATCC

GGAATCACTTCGGATGGATCATGATTAAATTGGCTAACGTCAGCGAATGATAAAACCCGCGGAATACACTCGGATGGATCGAAGTTAAACCCAGCCATCC

GGAATCACTTCGGATGGATCATGATTAAATTGGCTAACGTCAGCGAATGATAAAACCCGCGGAATACACTCGGATGGTTCGAAATTAAACCCAGCCCCCC

GGAATCACTGCGGATGGATCATGATTAAATTGACTAACGTCAGCGAATGACAAAACCTGCGGAATACACTCGGATGGATCGAAATTAAACCCAGACACCC

GGAATTACTTCGGATGGATCATGATTAAATTGGCTAACGTCAGCGAATGACAAAACCTGCGGAATACACTCGGATGGATCGAAGTTAAACCCAGCCATCC

GGAATCACTTCGGATGGATCATGATTAAATTGGCTAACGTCAGCGAATGATAAAACCCGCGGAATACACTCGGATGGATCGAAATTAAACCCAGACACCC

GGAATCACTTCGGACGGATCATGATTAAATTGGCTAACGTCAGCGAATGATAAAACCTGCGGAATACACTCGGATGGATCGAAGTTAAACCCAGCCATCC

GGAATCACTTCGGATGGATCGAAATTAAA------------------------------------------------------------CCCAGCCCCCC

GGAATAACTTCGGATGGATCATGATTAAATTGGCTAACGTCAGCGAATGATAAAACCTGCGGAATGCACTCGGATAGATCGAGGTTAAACCCAGCCATCC

----ATGGTACAAAGGTCGTGGCCAGATAAAGCAAAACACCACTCCATACACAGCCCG-GAGCGGCAA-TATAGCTGCAGTCGTCGCTAAACAATCACCCAC

----ATGCTACAAAGGTCGTGGCCAGATAAAGCAAAACACCACTCCATACACAGCCCG-GAACGGCAA-TATAGCTGCAGTCGTCGCTAAACAATCACCCAC

----ATGGTACAAAGGTCGTGGCCAGATAAAGCAAAACACCACTCCATACACAGCCCG-GAGCGGCAA-TATAGCTGCAGTCGTCGCTAAACAATCACCCAC

---CATGGTACAAAGGCCGTGCCCAGATAAAGCAAGACTCCACTCCATACACAGCCCGAGAGCGGAAA-TATAGCTGCAGTCATGGTTAAACAATCATCCAC

----ATGGTACAAAGGTCGTGCCCAGATAACGCAAGACTCCACTCCATACACAGCCCGAGAGCGGCAA-TATAGCTGCAGTCGTGGTTAAACAATCATCCAC

----ATGGTACAAAGATCGTGGTCAGATAAAGCAAGACTCCACTCCATACACAGCCCG-GAGCGGCAA-TATAGCTGCCGTCTTGGTTAAACAATCCCCCAC

--CCATGGTACAAAGGTCGTAGCCAGATAAAGCAAGACTCCACTCCATACACAGCCCGAGAGCGGCAAATATAGCTGCAGTCGTGGTTAAACAATCATCCAC

----ATGGTACAAAGGTCGTGGCCAGATAAAGCAAAACACCACTCCATACACAGCCCG-GAGCGGCAA-TATAGCTGCAGTCGTCGCTAAACAATCACCCAC

----ATGGTACAAAGGTCGCGACCAGATAAAGCAAGACTCCACTCCATACACAGCCCGAGAGCGGCAA-TATAGCTGCAGTCGTGGTTAAACAATCATCCAC

----ATGGTACAAAGATCGTGGCCAGATAAAGCAAAACACCACTCCATACACAGCCCG-GAGCGGCAA-TATAGCTGCAGTCATGGTTAAACAATCATCCAC

----ATGGTACAAAGGTCGCGGCCAGATAAAGCAAGACTCCACTCCATACATAGCCCGAGAGCGGCAA-TATAGCTGCAGTCGTGGTTAAACAATCATCCAC

CCCCATGGTACAAAGGTCGTAGCCAGATAAAGCAAGACTCCACTCCATATACAGCCCGAGAGCGGCAA-TATAGCTGCCATCTTGGTTAAACAATCACCCAC

----ATGGTACAAAGGTCGTGCCCAGATAAAGCAAGACTCCACTCCATACACAGCCCGAGAGCGACAA-TATAGCTGCAGTCTTGGTTAAACAATCACCCAC

GCGTGCC-TATATAGGCATATAGTGTTCAATTTAACCCGACAAGGCCATCGCTAACTACA-TGTACGCCACATCGCCCGGGGCATGCTCGGATGGATCGT

GCGTGCC-TATATAGGCATATAGTGTTCAATTTAACCCGACAAGGCCATCGCTAACTACA-TGTACGCCACATCGCCCGGGGCATGCTCGGATGGATCGT

GCGTGCC-TATATAGGCATATAGTGTTCAATTTAACCCGACAAGGCCATCGCTAACTACA-TGTACGCCACATCGCCCGGGGCATGCTCGGATGGATCAT

TCGGCTG-TATATAGGCATATAGCGCTCAATTTAGCCCAACAAGACCATCGCTAACTACA-TGTACGCCACATCGCGCGGGGCATGCTCGGATGGATCGT

TCGTGCTGTATATAGGCATATCGCGCTCAATTTAGTCCAATAAGGCCACCGCTAACCACAATGTACGCCACATCGCCCGGGGCATGCTCGGATGGATCGT

GCCTGCC-TATATAGGCATATAGCGCTCAATTTAGCCCAACAAGACCATCGCTAACTACA-TGTACGCCACATCGCCCGGGGCATGCTCGGATGGATCGT

TCGTGCTGTATATAGGCATATCGCGCTCAATTTAGCCCAACAAGGCCATCGCTAACCAAAATGTACGCCACATCGCGCGGGGCATGCTCGGATGGATCGT

GCGTGCC-TATATAGGCATATAGTGTTCAATTTAACCCAACAAGACCATCGCTAACTACA-TGTACGCTACATCGCTCGGGGCATGCTCGGATGGATCGT

GCGTGCC-CATATAGGCATATAGCGCTCAATTTAGCCCAACAAGACCATCGCTAACTACA-TGTACGCCACATCGCCCGGGGCATGCTCGGATGGATCGT

TCGTGCTGTATATAGGCATATAGCGCTCAATTTAGCCCAACAAGACCATCGCTAACTACA-TGTACGCCACATCGCGCGGGGCATGCTCGGATGGATCGT

TCGTGCTGTATATAGGCATATAGCGCTCAATTTAGCCCAACAAGACCATCGCTAACTACA-TGTACGCCACATCGCCCGGGGCATGCTCGGATGGATCGT

TCGTACTGTATATAGGCATATCGCGCTCAATTTAGTCCAATAAGGCCACCGCTAACCACAATATACGCCACATCGCCCGGGGCATGCTCGGATGGATCGT

TCGCACTGTATATAGGCATATCGCGCTCAATTTAGCCCAACAAGGCCATCGCTAACCACAATGTACGCCACATCGCCCGGGGCATGCTCGGATGGATCGT

CGCCAAATTGACGAATATTGCAGAACAAACAGGCGGAATAGTGTCGGATGGATCGCGATTAAATGGAGAGGGATAGGGAATACATCACATCTATATCAAG

CGCCAAATTGACGAATATTGCAGAACAAACAGGCGGAATAGTGTCGGATGGATCGCGATTAAATGGAGAGGGATAGAGAATACATCACATCTACATCAAG

CGCCAAATTGACGAATATTGCAGAACAAACAGGCGGAATAGTGTCGGATGGATCGCGATTAAATGGAGAGGGATAGAGAATACATCACATCTACATCAAG

CGCCAAATTGACGAATATTGCAGAATAAACAGGCGGAATAGTGTCGGATGGATCGCGATTAAATGGAGAGGGATAGGGAATACTTCACATCCACATCAAG

CGCCAAATTGACAAATATTGCAGAACAAACCGGCGGAATAGTGTCGGATGGATCGCGATTAAATGGAGAGGGATAGGGAATACATCACATCTATATCAAG

CGCCAAATTGACGAATATTGCAGAACAAACAGGCGGAATAGTGTCGGATGGATCGCGTTTAAATGAAGAGGGATAGAGAATACATCACATCTATATCAAG

CGCCAAATTGACGAATATAGCAGAACAAACAGGCGGAATAGTGTCGGATAGATCGCGATTAAATGGAGAGGGATAGGGAATACATCACATCTATATCAAG

CGCCAAATTGACGAATATTGCAGAACAAACAGGCGGAATAGTGTCGGATGGATCGCGATTAAATGGAGAGGGATAGAAAATACATCACATCTACATCAAG

CGCCAAATTGACGAATATTGCAGAACAAACAGGCGGAATAGTGTCGGATGGATCGCGATTAAATGGAGAGGGATAGGGAATACATAACATCTATATCAAG

CGCCAAATTGACGAATATTGCAGAACAAACAGGCGGAATAGTGTCGGATGGATCGCGATTAAATGGAGAGGGATAGGGAATACATCACATCCACATCAAG

CGCCAAATTGACGAATATTGCAGAACAAACAGGCGGAATAGTGTCGGATGGATCGCGATTAAATGGAGAGGGATAGGGAATACATCACATCTATATCAAG

CGCCAAATTGACGAATATTGCAGAATAAACAGGCGGAATAGTGTCGGATGGATCGCGATTAAATGGAGAGGGATAGGAAATACATCACATCTATATCAAG

CGCCAAATTGACGAATATTGCAGAACAAACAGGCGGAATAGTGTCGGATGGATCGCAATTAAATGGAGAGGGATAGAGAATACATCACATCTACATCAAG

CCCTCGCCATGACAAACCAGAGAAATGGGCTGATTTTAGCCATTATTTGCAAACAACTTCACATCGCC-TACGTGAAACCCACCAAGTTAAAAGTGAAAA

CCCTCGCCATGACAAACCAGAGAAATGGGCTGGCTTTAGCCATTATTTACAAACAACTTCACATTGCCATACGTGAAATTCACCAAGTTAAAACTAATAA

CCCTCGCCATGACAAACCAGAGAAATGGGCTGGCTTTAGCCATTATTTACAAACAACTTCACATTGCCATACGTGAAATTCACCAAGTTAAAACTAATAA

CCCTCGCCATGACAAACCAGAGAAATGGGCTGATTTTAGCCATTATTTACAAACAACTTCACATTGCCATACGTGAAATCCACCAAGTTAAAACAAATAA

CCCTCGCCATGACAAACCAGAGAAATGGGCTGATATTAGCCATTATTTACAAACAACTTCACATTGCCATACGTGAAATTCACCAAGTTAAAACTAATAA

CCCTCGCCATGACAAACCAGAGAAATGGGCTGATGTTAGCCATCATTTACAAACAACTTCACATAGTCATACGTGAAACCCACCAAGTTAAAACTAATAA

CCCTCGCCATGACAAAACAGAGAAATGGGCTGGTTTTAGCCATTATTTACAAACAACTTCACATTGCCATACGTGAAATTCACCAAGTTAAAACTAATAA

CCCTCGCCATGACAAACCAGAGAAATGGGCTGGCTTTAGCCATTATTTACAAACAACTTCACATTGCCATACGTGAAATTCACCAAGTTAAAACTAATAA

CCCTCGCCATGACAAACCAGAGAAATGGGCTGGTTTTAGCCATTATTTACAAAAAACTTCACATTGCCATACGTGAAATTCACCAAGTTAAAACTAATAA

CCCTCGCCATGACAAACCAGAGAAATGGGCTGGTTTTAGCCATTATTTACAAACAACTTCACATTGCCATACGTGAAATTCACCAATTTAAAAGTAAAAG

CCCTCGCCATGACAAACCAGAGAAATGGGCTGGTTTTAGCCATTATTTACAAACAACTTCACATTGCCATACGTGAAACTCACCAATTTAAAAGTAAAAG

CCCTCGCCATGACAAACCAGAGAAATGGGCTGATTTTAGCCATTATTTGCAAACAACTTCACATCGCC-TACGTGAAATCCACCAAGTTAAAAGTAAAAA

CCCTCGCCATGACAAACCAGAGAAATAGGCTGGCTTTAGCCATTATTTACAAACAACTTCACATTGCCATACGTGAAATTCACCAAGTTAAAACTAATAA

TAATTTTATAAAACAATACATTACCGTTATCAGAGTAACGTTAGCCATACTCGCCATCACATCAGATCGTGCGGAATAGCTACGGACGGATCGCAGTTAA

TA-TTTTATAACTCAATGCATTACTGTTATCAGAGTGACATTAACCATACTCGCCATCACATCAGATCGAGCGGAATAGCTACGGACGGATCGCAGTTAA

TA-TTTTATAACTCAATGCATTACTGTTATCAGAGTGACATTAACCATACTCGCCATCACATCAGATCGAGCGGAATAGCTACGGACGGATCGCAGTTAA

TT-ACTTATAGATCAATGTATTGCAGTTATCAGAGTGACATTAGCTATGCTCGTCATCACATCAGATCGTGCGGAATAGCTACGGACGGATCGCAGTTAA

TTATCT-ATAGATCAATATATTGCAGTTATCAGAGTGACATTAGCCATGCTCGTCATCACATCAGATCGTGCGGAATAGCTACGGACGGATCGCAGTTAA

TT-ATTTATAGATCAATATATTGCAGTTATCAGAGTGACATTAGCTATACTCGTCATCACATCAGATCGTGCGGAATAGCTACGGACGGATCGCAGTTAA

TT-ATTTATAGATCAATATATTGCAGTTATCAGAGTGACATTAGCTATGCTCGTCATCACATCAGATCGCGCGGAATAGCTACGGACGGATCGCAGTTAA

TA-TTTTATAACTCAATGCATTACTGTTATCAGAGTGACATTAACCATACTCGCCATCACATCAGATCGAGCGGAATAGCTACGGACGGATCGCAGTTAA

TT-ATTTATAGATCAATATATTGCAGTTATCAGAGTGACATTAGCCATGCTCGTCATCACATCAGATCGTGCGGAATAGCTACGGACGGATCGCAGTTAA

TATTTTTATAAAACAATACATTACCGTTATCAGAGTGACATTAACCATACTCGCCATCACATCAGATCGTGCGGAATAGCCACGGACGGATCGCAGTTAA

TATTTTTATAAAACAATACATTACCGTTATCAGAGTGACATTAACCATACTCGCCATCACAACAGATCGTGCGGAATAGCCACGGACGGATCGCAGTTAA

TAATTTTATAAAACAATATATTACTGTTATAAGAGTAACGTTAGCCATACTCGCCATCACATCAGATCGTGCGGAATAGCTACGGACGGATCGCAGTTAA

TA-TTTTATAACTCAATGCATTACTGTTATCAGAGTGACATTAACCATACTCGCCATCACATCAGATCGAGCGGAATAGCTACGGACGGATCGCAGTTAA

AATAACGCTCAAAATCATCAGCACATCACACCGCACAAAAGATCCAGCCGCTGATATACACCGATCCTTCCGCTAACACATCGAACAGCCATCACGCTTT

AATAACACTCAAAATCATCAGCGGATCACACCGCGCACAAGATCCTGCCGCGGATATACACCGCTCCCTCCGCTAACACATCGAACAGCCACCACACTTT

AATAACACTCAAAATCATCAGCGGATCACACCGCGCACAAGATCCTGCCGCGGATATACACCGATCCCTCCGCTAACACATCGAACAGCCACCACACTTT

AATAACGCCCAAAATCATCAGCGGATCACACCGCGCACAAGATCCTTCCGCGGATATACACCGATCCCTCCGCTAACACATCGA-CGGCCACCACGCTTT

AATAACGCTCAAAATCATCAGCGGATCACGCCACGCACAAGATCCTGCCGCGGATATACACCGATCCTTCCGCTAACACATCGAACAGCCATCACGCTTT

AATAACACTCAAAATCATCAGCGGATCACACCGCGCACAAGATCCTGCCGCGGATATACACCGCTCCCTCCGCTAACACATCGAACAGCCACCACACTTT

AATAATGCCCAAAATCATCAGCGGATCACACCGCGCACAAGATCCTGCCGCTGATATATACCAATCCCTCCGCTAACACATCGAACAGGCACCACGCTTT

AATAACACTCAAAATCATCAGCGGATCACACCGCGCACAAGATCCTGCCGCGGATATACACCGCTCCCTCCGCTAACACATCGAACAGCCACCACACTTT

AATAACGCTCAAAATCATCAGCGGATCACCCCGCGCAAAAGATCCTGCCGCGGATATACACCGATCCCTCCGCTAACACATCGAACGGCCACCACGCTTT

AATAACGCTCAAAATCATCAGCGGCTCACTCCGCGCACAAGATCCTGCCGCAGATATGCACCGATCCCTCCGCTAACGCACCGAACGGCCACCACGCTTT

AATAACGCTCAAAATCATCAGCGGCTCACTCCGCGCACAAGATCCTGCCACAGATATGCACCGATCCCTCCGCTAACGCACCGAACGGCCACCACGCTTT

AATAACGCTCAAAATCATCAGCGGATCACCCCGCGCAAAAGATCCTGCCGCGGATATACACCGATCCCTCCGCTAACACATCAAACAGGCACAACGCTTT

AATAACACTCAAAATCATCAGCGGATCACACCGCGCACAAGATCCTGCCGCGGATATACACCGATCCCTCCGCTAACACATCGAACAGCCACCACACTTT

GCACGCAACCAGACGCATAATCGCGCCAAACACCGGCAAAAATGCGACCGCCATCAAATCCGCCGGTTCAATCAGCAATGACTTTGAAATTAAAAATGTC

GCACGCAACCAGAGGCATAATCGCGCCAATCACCGGTAAAAATGCGACCGCCATCAAATCCGCCGGTTCAATCAGCAATGACTTTGAAATTAAAAATGTC

GCACGCAACCAGACGCATAATCGCGCCAATCACCGGTAAAAATGCGACCGCCATCAAATCCGCCGGTTCAATCAGCAATGACTTTGAAATTAAAAATGTC

GCACGCAACCAGAGGCATAATCGCACCAATCACCGGCAAAAATGCGACCGCCATCAAATCCGCCGGTTCAATCAGCAATGACTTTGAAATTAAAAATGTC

GCACGCAACCAGACGCATAATCGCGCCAATCACCGGTAAAAATGCGACCGCCATCAAATCCGCCGGTTCAATCAGCAATGACTTTGAAATTAAAAATGTC

GCACGCAACCAGAGGCATAATCGCGCCAATCACCGGTAAAAATGCGACCGCCATCAAATCCGCCGGTTCAATCAGCAATGACTTTGAAATTAAAAATGTC

GCACGCAACCAGACGCATAATCGCGCCAAACACCGGCAAAAATGCGACCGCCATCAAATCCGCCGGTTCAATCAGCAATGACTTTGAAATTAAAAATGTC

GCACGCAACCAGAGGCATAATCGCGCCAATCACCGGTAAAAATGCGACCGCCATCAAATCCGCCGGTTCAATCAGCAATGACTTTGAAATTAAAAATGTC

GCACGCAACCAGACGCATAATCGCGCCAATCACCGGCAAAAATGCGACCGCCATCAAATCCGCCGGTTCAATCAGCAATGACTTTGAAATTAAAAATGTC

GCACGCAACCAGACGCATAATCGCGCCAATCACCGGCAAAAATGCGACCGCCATCAAATCCGCCGGTTCAATCAGCAATGACTTTGAAATTAAAAATGTC

GCACGCAACCAGAGGCATAATCGCGCCAATCACAGGCAAAAATGCGACCGCCATCAAATCCGCCGGTTCAATCAGCAATGACTTTGAAATTAAAAATGTC

GCACGCAACCAGACGCATAATCGCGCCAAACACCGGCAAAAATGCGACCGCCATCAAATCCGCCGGTTCAATCAGCAATGACTTTGAAATTAAAAATGTC

GCACGCAACCAGACGCATAATCGCGCCAATCCCCGGTAAAAATGCGACCGCCATCAAATCCGCCGGTTCAATCAGCAATGACTTTGAAATTAAAAATGTC

ACACCGGCGGAGCAACTGCTAGACTACGCCGGTAACACCTAGCAACAAGCTCGCAGATCGCGTTTAATTGATGAAAAAAA-CAGCGTTATTCACACCCAT

ACACCGGCGGAGCAACTGCTAGACTACGCCGGTAACACCTAGCAACAAGCTCGCAGAGCGCGTTTGATTGACGAAAAAAA-CAGCGTTATTCACACCCAT

ACACCGGCGGAGCAACTGCTAGACTACGCCGGTAACACCTAGCAACAAGCTCGCAGAGCGCGTTTGATTGACGAAAAAAA-CAGCGTTATTCACACCCAT

ACACCGGCGGAGCAACTGCTAGACTACGCCGGTAACGCCTAGCAACAAGCTCTCAGAGCGCGTTTGATTGACGAAAAAAA-CAGCGTTATTCACACCCAT

ACACCGGCGGAGCAACTGCTAGACTACGCCGGTAACACCTAGCAACAAGCTCGCAAAGCGCGTTTGATTGACGAAAAAA--CAGCGTTATTTACACCCAT

ACACCGGCGGAGCAACTGCTAGACTACGCCGGTAACACCTAGCAACAAGCTCGCTGAGCACGTTTGATTGACGAAAAAA--CAGCGTTATTCACACCCAT

ACACCGGCGGAGCAACTGCTAGACTAGGCCGGTAACACCTAGCAACAAGCTCGCAAAGCGCGTTTGATTGACGAAAAAAA-CAGCGTTATTCACACCCAT

ACACCGGCGGAGCAACTGCTAGACTACGCCGGTAACACCTAGCAACAAGCTCGCAGAGCGCGTTTGATTGACGAAAAAAA-CAGCGTTATTCACACTCAT

ACACCGGCGGAGCAACTGCTAGACTACGCCAGTAACACCTAGCAACAAGCTCGCAGATCGCGTTTGATTGACGAAAAAAA-CAGCGTTATTCACACCCAT

ACACCGGCGGAGCAACTGCTAGACTACGCCGGTAACAC-TAACAACAAGCTCGCAGAGCGCGTTTGATTGACGAAAAAAA-CAGCGTTACTCACATCCAT

ACACCGGCGGAGCAACTGCTAGACTACGCCGGTAACACCTAGCAACAAGCTCGCAGAGCGCGTTTAATTGACGAAAAAAA-CAGCGTTATTCACACCCAT

ACACCGGCGGAGCAACTGCTAGACTACGCCGGTAACACCTAGCAACAAGCTCGCAGAGCGCGTTTGATTGACGAAAAAAAACAGCGTTATTCACACCCAT

ACACCGGCGGAGCAACTGCTAGACTACGCCGGTAACACCTAGCAACAAGCTCGCAGATCGCGTTTGATTGACGAAAAAAA-CAGCGTTATTCACACCCAT

ATCGCAGCGTGAAACATGGCAGCCGCCGCCAAGGTGAAAAGGTTGGACACCGTCGTCAATAACCTGTTTACAGGCTGTGATACTTACGAATGGATCGTTT

ATCGCACCTTGGAACATGGCAGCCGCCGCCAAGGTGAAAAGGTTGGACACCGTCGTCAATAACCTGTTTACAGGCTGTGATACTTACGAATGGATCGTTT

ATCGCACCTTGGAACATGGCAGCCGCCGCCAAGGTGAAAAGGTTGGACACCGTCGTCAATAACCTGTTTACAGGCTGTGATACTTACGAATGGATCGTTT

ATCGCAGTGTGAAACATGGCAGCAGCCGCCAAGGTGAAAAGGTTGGACACCGTCGTCAATAACCTGTTTATAGGCTGTGATACTTACGAATGGATCGTTT

ATCGCACCTTGGAACATGGCAGCTGCCGCCAAGGTGAAAAGCTTGGATACCGTCGTCAATAACCTGTTTACAGGCTGTGATACTTACGAATGGATCGTTT

ATCGCAGCGTGAAACATGGCAGCCGCCGCCAAGGTGAAAAGCTTGGATACCGTCGTCAATAACCTGTTTACAGGCTGTGATACTTACGAATGGATCGTTT

ATCGCAGCGTGAAACATGGCAGCCGCCGCCAAGGTGAAAAGGTTGGACACCGTCGTCAATAACCTGTTTACAGGCTGTGATACTTACGAATGGATCGTTT

ATCGCAGCGTGAAACATGGCAGCCGCCGCCAAGGTGAAAAGGTTGGACACCGTCGTCAATAACCTGTTTACAGGCTGTGATACTTACGAATGGATCGTTT

ATCGCAGCGTGATACATGGCAGCCGCCGCCAAGGTGAAAAGGTCGGACACCGTCGTCAATAACCTGTTTACAGGCTGTGATACTTACGAATGGATCGTTT

ATCGCACCTTGGAACATGGCAGCCGCCGCCAAGGTGAAAAGGTTGGACACCGTCGTCAATAACCTGTTTACAGGCTGTGATACTTACGAATGGATCGTTT

ATCGCACCTTGGAACATGGCAGCCGCCGCCAAAGTGAAAAGGTTGGACACCGTCGTCAATAACCTGTTTACAGGCTGTGATACTTACGAATGGATCGTTT

ATCGCACCTTGGAACATGGCAGCCGCCGCCAAGGTGAAAAGGTTGGACACCGTCGTCAATAACCTGTTTACAGGCTGTGATACTTACGAATGGATCGTTT

ATCGCAGCGTGAAACATGGCAGCCGCCGCCAAGGTGAAAAGGTGGGACACCTTCGTCAATAACCTGTTTACAGGCTGTGATACTTACGAATGGATCGTTT

TTAACGTACGGTGGATCGTTCAAGGAACAGAAGTGGATCGTCAGTTAAACAACGCGTGGATCGTTGCTAACAAAGGGTGGATCGTTAACTAAACAGGCGG

TTAACGTAGCGTGGATCGTTCAAGGAACAGAAGTGGATCGTCAGTTAAACAACGCGTGGATCGTTGCTAACAAAGGGTGGATCGTTAACTAAACAGGCGG

TTAACGTAGCGTGGATCGTTCAAGGAACAGAAGTGGATCGTCGGTTAAACAACGCGTGGATCGTTGCTAACAAAGGGTGGATCGTTAACTAAACAGGCGG

TTAACGTAGTGTGGATCGTTCAAGGAACAGAAGTGGATCGTCAGTTAAACAACGCGTGGATCGTTGCTAACAAAGGGTGGATCGTTAACTAAATAGGCGA

TTAACGTAGCGTGGATCGTTCAAGGAACAGAAGTGGATCGTCAGTTAAACAACGCGTGGATCGTTGCTAACAAAGGGTGGATCGTTAACTAAACAGGCGG

TTAACATAGCGTGGATCGTTCAAGGAACAGAAGTGGATCGTCAGTTAAACAACGCGTGGATCGTTGCTAACAAAGGGTGGATCGTTAACTAAACAGGCGG

TTAACGTAGTGTGGATCGTTCAAGGAACAGAAATGGATCGTCAGTTAAACAATGCGTGGATCGTTGCTAACAAAGGGTGGATCGTTAACTAAACAGGCGG

TTAACGTAGCGTGGATCGTTCAAGGAACAGAAGTGGATCGTCAGTTAAACAACGCGTGGATCGTTGCTAACAAAGGGTGGATCGTTAACTAAACAGGCGG

TTAACGTAGGGTGGATCGTTCAAGGAACAGAAGTGGATCGTCAGTTAAACAACGCGTGGATCGTTGCTAACAAAGGGTGGATCGTTAACTAAACAGGCGG

TTAACGTAGCGTGGATCGTTCAAGGAACAGAAGTGGATCGTCGGTTAAACAACGCGTGGATCGTTGCTAACAAAGGGTGGATCGTTAACTAAACAGGCGG

TTAACGTAGGGTGGATCGTTCAAGGAACAGAAGTGGATCGTCAGTTAAACAACGCGAGGATCGTTGCTAACAAAGGGTGGATCGTTAACTAAACAGGCGG

TTAACGTAGCGTGGATCGTTCAAGGAACAGAAGTGGATCGTCGGTTAAACAACGCGTGGATCGTTGCTAACAAAGGGTGGATCGTTAACTAAACAGGCGG

TTAACGTAGGGTGGATCGTTCAAGGAACAGAAGTGGATCGTCAGTTAAACAACGCGTGGATCGTTGCTAACAAAGGGTGGATCGTTAACTAAACAGGCGG

TTTTTATTTGGATCGTAGTTTTTAATGCATTGAATAACTTAGCTTTTTAACGGTTTCCCACAGTACCCATGATCTAAGATAAATAAGATATAAAGAAAAA

TTTTTATTTGGATCGCAGTTTTTAATGTATTGAATAACTTAGCTTTTTAACGGTTACCCACAGTACCCATGATCTAAGATAAGTAAGATATAAAGAAAAA

TTTTTATTTGGATCGCAGTTTTTAATATATTGAATAACTTAACTTTTTAACGGTTACCCACAGTACCCATGATCTAAGATAAATAAGATATAAAGAAAAA

TTTTTATTTGGATCGCAGTTTTTAATATATTGAATAACTTAACTTTTTAACGCTTACCCACAGTACCCATGATCTAAGATAAATAAGATATAAAGAAAAA

TTTTTATTTGGATCGCAGTTTTTAATGTATTGAATAAATTAGCTTTTTAACGGTTACCCACAGTACCCATGATCTAAGATAAATAAGATATAAAGAAAAA

TTTTTATTTGGATCGCAGTTTTTAATATATTGAATAACTTAGCTTTTTAACGGTTACCCACAGTACCCATGATCTAAGATAAATAAGATATAAAGAAAAA

TTTTTATTTGGATCGCAGTTTTTAATGCATTGAATAACTTAGCTTTTTAACGGTTACCCACAGTACCCATGATCTAAGATAAATAAGATATAAAGAAAAA

TTTTTATTTGGATCGCAGTTTTTAATGTATTGAATAACTTAGCTTTTTAACGGTTACCCACAGTACCCATGATCTAAGATAAGTAAGATATAAAGAAAAA

TTTTTATTTGGATCGCAGTTTTTAATGCATTGAATAACTTAGCTTTTTAACGGTTACCCACAGTACCCATGATCTAAGATAAATAAGATATAAAGAAAAA

TTTTTATTTGGATCGCAGTTTTTAATATATTGAATAACTTAACTTTTTAACGGTTACCCACAGTACCCATGATCTAAGATAAATAAGATATAAAGAAAAA

TTTTTATTTGGATCGCTGTTTTTAATATATTGAATAACTTAACTTTTTAACGGTTACCCACAGTACCCATGATCTAAGATAAGTAAGATATAAAGAAAAA

TTTTTATTTGGATCGCAGTTTTTAATATATTGAATAACTTAACTTTTTAACGGTTACCCACAGTACCCATGATCTAAGATAAATAAGATATAAAGAAAAA

TTTTTATCTGGATCGCAGTTTTTAATTTATTGAATAACTTAGCTTTTTAACGGTTACCCACAGTACCCATGATCTAAGATAAATAAGATATAAAGAAAAA

AA-GAATCAGGATATAAACAAACAATAAGTTTTTTT-GGATTTCTAAAATG

*rctB*

AA-GAATCAGGATATAAACAAACAATAAGTTTTTTT-GGATTTCTAAAATG

AAAGAATCAGGATATAAACAAACAATAAGTTTTTTT-GGATTTCTAAAATG

AA-GAATCAGGATATAAACAAACAATAAGTTTTTTT-GGATTTCTAAAATG

AA-GAATCAGGATATAAACAAACAATAAGTTTTTTT-GGATTTCTAAAATG

AA-GAATCAGGATATAAACAAACAATAAGTTTTTTT-GGATTTCTAAAATG

AA-GAATCAGGATATAAACAAACAATAAGTTTTTTT-GGATTTCTAAAATG

AA-GAATCAGGATATAAACAAACAATAAGTTTTTTT-GGATTTCTAAAATG

A--GAATCAGGATATAAACAAACAATAAGTTTTTTT-GGATTTCTAAAATG

AA-GAATCAGGATATAAACAAACAATAAGTTTTTTT-GGATTTCTAAAATG

AA-GAATCAGGATATAAACAAACAATAAGTTTTTTT-GGATTTCTAAAATG

AA-GAATCAGGATATAAACAAACAATAAGTTTTTTT-GGATTTCTAAAATG

AA-GAATCAGGATATAAACAAACAATAAGTTTTTTTTGGATTTCTAAAATG

10 mer 8 mer

AGCCTCGGACGGATCGCAAT CGAATGGATCGTTTTT

CACTTCGGATGGATCATGAT ACGGTGGATCGTTCAA

ACACTCGGATGGATCGAAAT GAAGTGGATCGTCAGT

ATGCTCGGATGGATCGTCGC CGCGTGGATCGTTGCT

AGTGTCGGATGGATCGCGAT AGGGTGGATCGTTAAC

AGCTACGGACGGATCGCAGT TATTTGGATCGTAGTT

NNNNTCGGAYGGATCGNNNN NNNNTGGATCGTNNNN
