# Supplementary figures and images for: Plesiomonas shigelloides, an Atypical Enterobacterales with a Vibrio-Related Secondary Chromosome
Source: Genome Biol Evol. 2022 Jan 25;14(2):evac011. doi: 10.1093/gbe/evac011 (PMC8826520; doi:10.1093/gbe/evac011)

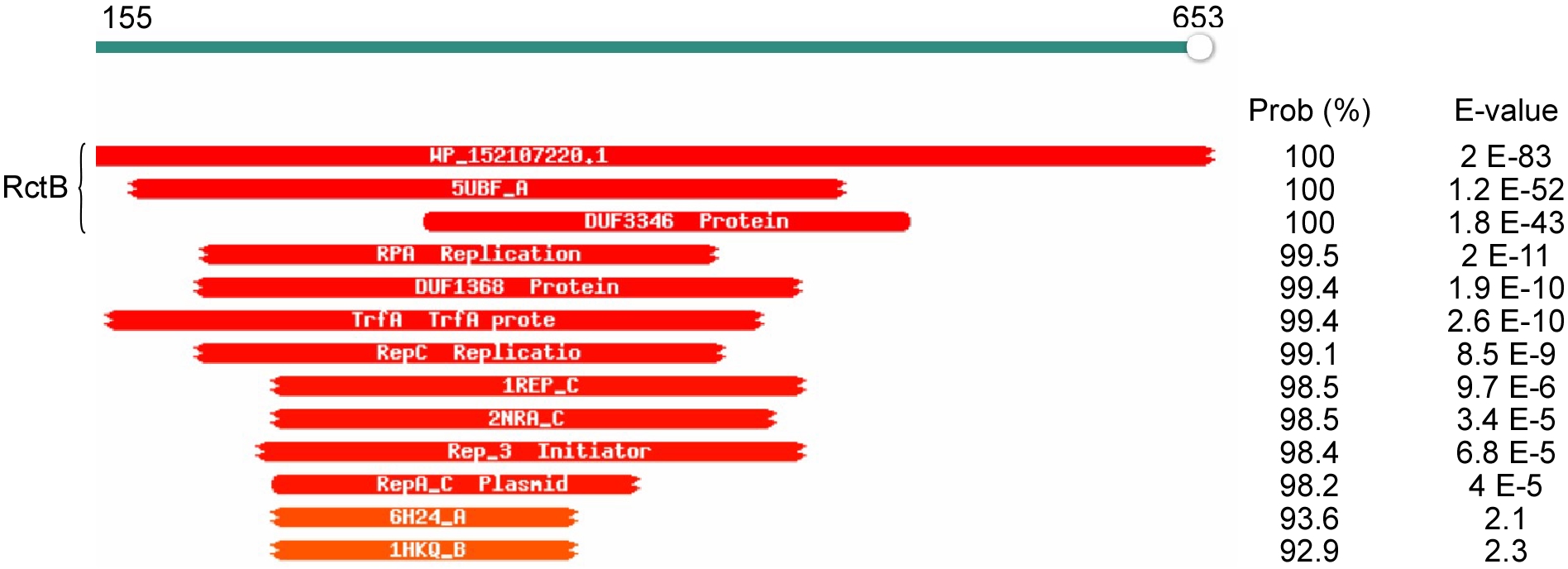

Supplement: evac011_Supplementary_Data [file evac011_supplementary_data.zip › sup_Figure 1.jpg]

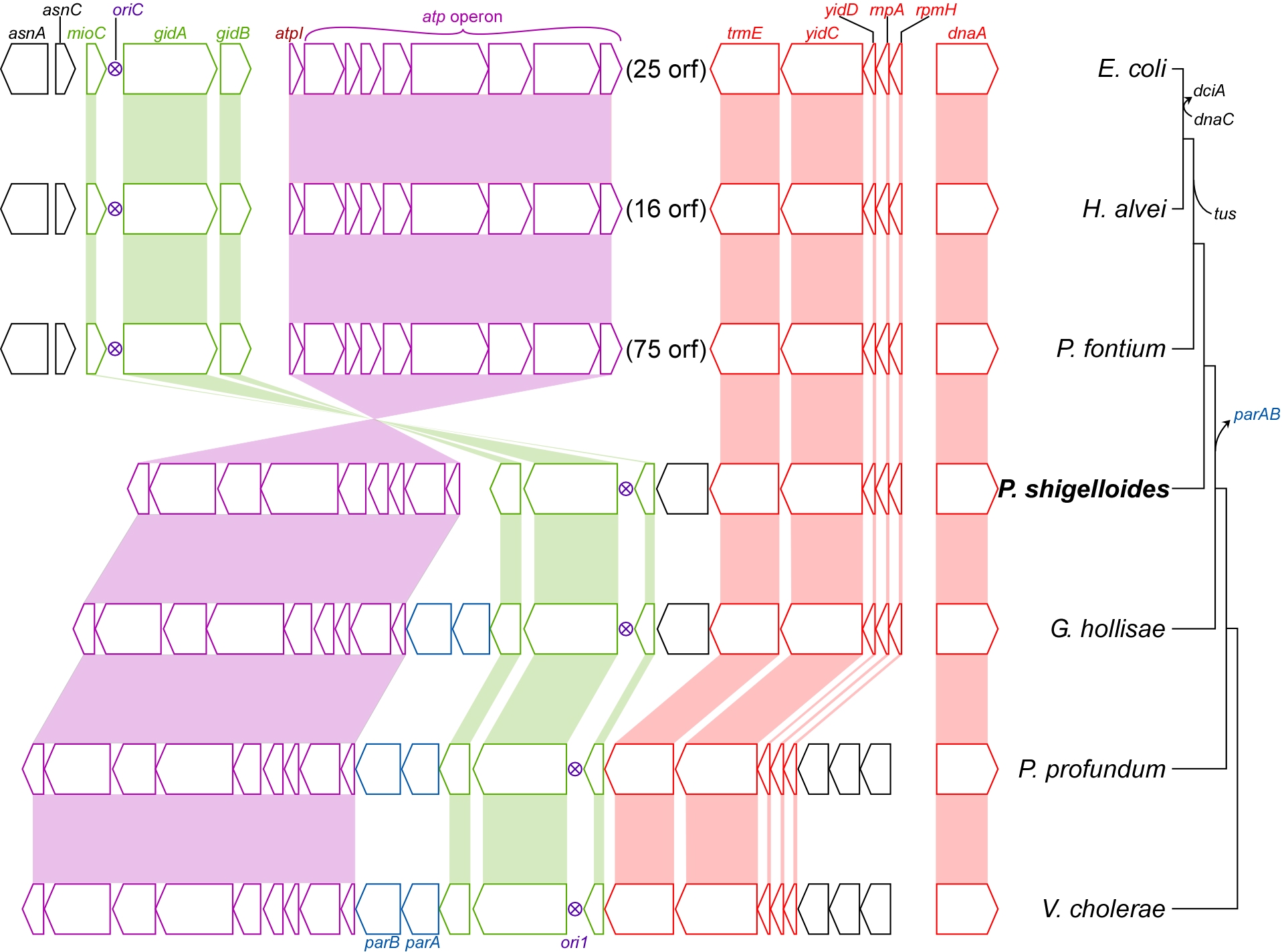

Supplement: evac011_Supplementary_Data [file evac011_supplementary_data.zip › sup_Figure 3.jpg]
